# Single-cell profiling unveils nephritis-related circulating immunological signatures in systemic lupus erythematosus patients

**DOI:** 10.1038/s42003-025-09431-8

**Published:** 2026-01-05

**Authors:** Qun Liu, Linjie Wu, Sha Hao, Yiyao Deng, Xiaomin Liu, Shunlai Shang, Yena Zhou, Jie Zhang, Qinggang Li, Ping Li, Ying Zheng, Xueyuan Bai, Xu Wang, Xiaowei Xie, Chaomin Guo, Liuyang Yang, Huayu Lin, Guangyan Cai, Tao Cheng, Xiangmei Chen

**Affiliations:** 1 School of Medicine, Nankai University, Tianjin, 300071 China; 2 Department of Nephrology, First Medical Center of Chinese PLA General Hospital, State Key Laboratory of Kidney Diseases, National Clinical Research Center for Kidney Diseases, Beijing Key Laboratory of Medical Devices and Integrated Traditional Chinese and Western Drug Development for Severe Kidney Diseases, Beijing Key Laboratory of Digital Intelligent TCM for the Prevention and Treatment of Pan-vascular Diseases, Key Disciplines of National Administration of Traditional Chinese Medicine(zyyzdxk-2023310), Beijing, 100853 China; 3 State Key Laboratory of Experimental Hematology, National Clinical Research Center for Blood Diseases, Haihe Laboratory of Cell Ecosystem, Institute of Hematology & Blood Diseases Hospital, Chinese Academy of Medical Sciences & Peking Union Medical College, Tianjin, 300020 China; 4 Tianjin Institutes of Health Science, Tianjin, 301600 China; 5 Department of Nephrology, Guizhou Provincial People’s Hospital, 83, Zhongshan Road, Nanming District, Guiyang, 550002 Guizhou China; 6 Department of Nephrology, China–Japan Friendship Hospital, Beijing, 100029 China; 7 Laboratory Medicine Department, First Medical Center of Chinese PLA General Hospital, Beijing, 100853 China; 8 Department of Blood Transfusion Medicine, The First Medical Center, Chinese PLA General Hospital, Beijing, 100853 China

**Keywords:** Lupus nephritis, Autoimmunity

## Abstract

Lupus nephritis (LN), the most severe complication of systemic lupus erythematosus (SLE), arises from systemic immune dysregulation and renal damage. While renal immune perturbations are well-studied, systemic signatures specific to LN pathogenesis remain unclear. Integrated single-cell RNA and immune repertoire analysis of 177,259 peripheral blood mononuclear cells (PBMCs) from healthy donors and SLE patients (including active LN and non-nephritis controls) revealed LN-specific circulating immune signatures, including κ light-chain preference in naive B cells and distinct clonal expansion in CD8^+^ effector T cells. These clonally expanded CD8^+^ effector T cells exhibited transcriptional variations indicating increased migratory capacity and exhaustion, along with preferential usage of TRBV genes (*TRBV27/TRBV15/TRBV7-9*), which have enhanced binding potential to an EBV epitope GLCTLVAM. Based on these findings, we developed a dual-biomarker model demonstrating reliable LN diagnosis (AUC = 0.895). Cross-tissue analysis confirmed concordance between peripheral and intrarenal immune perturbations, supporting non-invasive blood-based monitoring. Enhanced MIF-(CD74 + CXCR4) axis activity linked to lymphocyte activation/migration, while CD74^+^ memory B cells upregulated MHC-I antigen presentation. Renal immunostaining revealed CD74^+^ B cells proximal to CD8^+^ T cell infiltrates, suggesting CD74-mediated crosstalk facilitates intrarenal T cell activation. This study provides an integrated LN immune atlas, identifies translatable biomarkers and highlights CD74 as a potential therapeutic target.

## Abstract (web summary)

Integrated single-cell RNA and immune repertoire analysis of PBMCs identifies key circulating immune perturbations in lupus nephritis, highlighting non-invasive biomarkers and CD74 as a potential therapeutic target.

## Introduction

Systemic lupus erythematosus (SLE), a multisystemic autoimmune disorder, is characterized by dysregulated immune activation leading to heterogeneous organ damage^1–3^. Renal involvement (lupus nephritis, LN) is widely recognized as one of the most serious complications of SLE, exhibiting a greater prevalence and severity among Asian populations compared to those of Western European ancestry^4,5^. Current diagnostic reliance on invasive kidney biopsy—the clinical gold standard—highlights the urgent need for non-invasive biomarkers capable of reflecting renal involvement^6^. As the primary biological source for immune surveillance, peripheral blood has become the cornerstone for investigating immune dysregulation in autoimmune diseases^7^. Emerging evidence suggests that clonally expanded T and B cells found in the kidneys can also be identified in peripheral circulation, positioning blood-based immune profiling as a promising strategy for monitoring LN progression^8,9^.

LN development involves multiple cell types through immunological and non-immune processes^10^. A large-scale transcriptomic analysis of sorted peripheral immune subsets in SLE has uncovered cell-type-specific signatures linked to disease heterogeneity, implicating neutrophils, monocytes, Th1 cells, and plasmablasts as key mediators of renal involvement^11^. Recent advancements in single-cell technologies enable the unbiased characterization of diverse immune cell subsets without the need for prior sorting, while also facilitating the analysis of their interaction networks^12^.

Notably, single-cell RNA sequencing (scRNA-seq) studies of LN renal biopsies by Arazi et al. identified extensive expression of CXCR4-CX3CR1 across immune subclusters, implying their probable role in orchestrating leukocyte trafficking to inflamed kidneys^13^. Complementary to these findings, a recent study highlighted pathogenic lymphocyte interactions within the renal niche, specifically a coordinated intrarenal extrafollicular B cell response alongside hyperactivated GZMK^+^ CD8^+^ T cells in LN renal biopsies, proposing a local immune axis driving renal damage^14^. Despite these advances in characterizing intrarenal immunity, several important questions remain unresolved, including whether circulating immune cells in LN patients exhibit distinct molecular signatures compared to those in non-nephritic SLE patients; to what extent such peripheral alterations reflect the pathological immune signatures found in the renal microenvironment; and whether peripheral immune alterations can reliably reflect the immune dynamics within the kidney.

To bridge this knowledge gap, we performed integrated scRNA-seq and paired T/B cell receptor repertoire sequencing analyses on peripheral blood mononuclear cells (PBMCs) from SLE patients, with and without LN. By cross-referencing our findings with published renal scRNA-seq datasets, we identified pronounced concordance between circulating and intrarenal immune perturbations. Our integrative analysis uncovers nephritis-specific immune alterations with biomarker potential, establishes a composite logistic regression model for LN diagnosis, and reveals enhanced intercellular communication networks in LN patients, highlighting potential therapeutic targets. These findings collectively advance our understanding of LN immunobiology while providing clinically translatable tools for non-invasive monitoring and targeted intervention.

## Results

### Dramatic remodeling of circulating immune cells in lupus patients

**Fig. 1:**
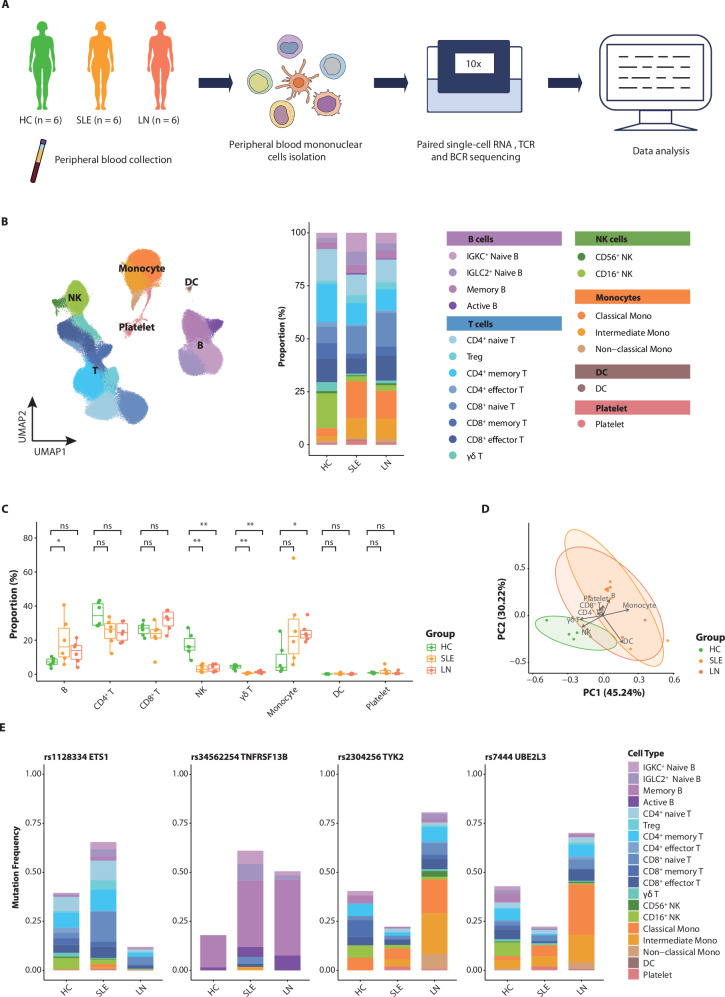
Overview of the single-cell transcriptome landscape of circulating immune cells in lupus patients. **A** Schematic showing sample collections and sequencing strategy for the discovery cohort (*n* = 6 per group). **B** Uniform manifold approximation and projection (UMAP) visualization for the discovery cohort. Bar plot shows the composition of peripheral blood mononuclear cells across systemic lupus erythematosus (SLE), lupus nephritis (LN), and healthy control (HC) groups (*n* = 6 per group). **C** Box plots illustrate proportions of major cell types across SLE, LN, and HC groups in the discovery cohort. Box: interquartile range (IQR); line: median; whiskers: 1.5 × IQR range from the quartiles; dots: individual samples (*n* = 6 per group). Statistical significance of the differences between patients and HC was determined using the Mann–Whitney *U* test (*P < 0.05, ***P < 0.001; *n* = 6 per group). **D** Principal component analysis (PCA) biplot of cell type compositions in the discovery cohort (*n* = 6 per group). The first two principal components (variance explained indicated in parentheses) are shown. Samples are represented as dots colored by group, and arrows indicate the direction and influence of specific cell types on the component loadings. **E** Mutation frequency of four reported SLE-related SNPs detected in the discovery cohort (*n* = 6 per group). Each bar represents the frequency of mutated cells among those with reads covering the respective loci, and the bars are color-coded according to the cell types.

To systematically characterize immune cell dynamics during lupus pathogenesis, we performed integrated analysis of scRNA-seq and immune repertoire profiling data from PBMCs of six SLE patients without nephritis (hereinafter referred to as SLE), six LN patients, and six HCs (Fig. 1A; samples P1–P12 and HC1–HC6; Supplementary Tables 1 and 2). To improve B cell annotation resolution—motivated by the central role of B lymphocytes in lupus pathogenesis—we additionally incorporated publicly available scRNA-seq data of CD19⁺ B cell-enriched PBMC samples from a previous lupus study^15^ (samples P13–P15 and HC7–HC9; dataset GSE193867). Following stringent quality control, 177,259 cells were clustered into 19 clusters through unsupervised clustering (Fig. 1B; Supplementary Fig. 1A–C, Supplementary Table 3, Supplementary Data 1). Comparative analysis revealed significant differences in the composition of circulating immune cells between patient groups and HCs, while the SLE and LN groups exhibited relatively conserved profiles. In lupus patients, the proportions of B cells and monocytes were markedly elevated, whereas the proportions of NK cells and γδ T cells were significantly lower than those in HCs (Fig. 1C; Supplementary Fig. 1D and E, Supplementary Data 2). Notably, consistency in immune cell composition was observed across lupus patients, especially among innate immune cell types (Fig. 1D).

To confirm the reliability of our dataset, we implemented a dual validation strategy. First, we calculated interferon-stimulated gene (ISG) scores using established signatures, revealing a significant elevation in SLE and LN groups compared to HCs. This activation was particularly prominent in monocytes—key innate immune responders—consistent with the recognized centrality of type I interferon signaling in lupus pathogenesis (Supplementary Fig. 1F). Second, we evaluated mutation frequencies at four known lupus-associated loci^16^ (Fig. 1E). The *TNFRSF13B* variant (rs34562254) exhibited significantly higher mutation rates in B cells from SLE and LN groups compared to HCs, with the most pronounced enrichment observed in the mature/activated B cell subclusters of LN patients. Functionally, *TNFRSF13B* encodes TACI, a receptor that governs B cell maturation through APRIL/BAFF interactions^17,18^. Its altered mutation frequency aligns with enhanced B cell activation profiles in lupus patients, particularly in those with LN. Moreover, monocytes demonstrated an increased *TYK2* variant (rs2304256) frequency in LN patients relative to both the SLE and HC groups. As *TYK2* (tyrosine kinase 2) is a critical component of the JAK-STAT pathway that regulates type I interferon responses^19,20^, its mutation pattern suggests a potential mechanistic involvement in the LN progression. These concordant genetic observations—aligning with both established disease pathways and our cellular subset proportion changes—collectively reinforce the biological validity of our dataset.

### Profiling of naive and active B cells unveiling crucial lupus-specific variations

**Fig. 2:**
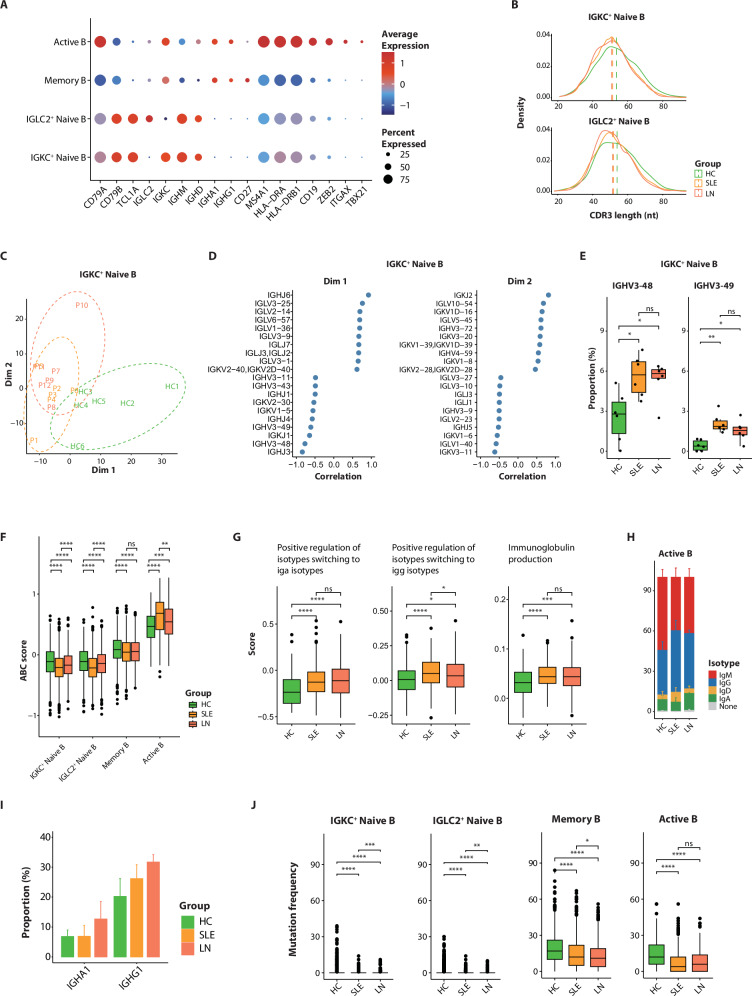
Alterations of circulating B cells in LN patients. **A** Dot plot illustrating the expression of marker genes in each B cell subset. The colors on the plot represent the average expression of the marker genes, while the size of the dots indicates the proportion of cells expressing these genes. **B** Heavy-chain CDR3 length distribution in naive B cell subclusters from the discovery cohort. **C** Principle component analysis of V/J gene usages in IGKC^+^ naive B cell subcluster from the discovery cohort (*n* = 6 per group). **D** Top 10V/J genes correlated with principal components in IGKC^+^ naive B cell subcluster. **E** The boxplots show IGHV genes exhibiting significantly increased usage in IGKC^+^ naive B cells of lupus patients within the discovery cohort. Box: IQR; line: median; whiskers: 1.5 × IQR range from the quartiles; dots: individual samples (*n* = 6 per group). Statistical significances were determined using the Mann–Whitney *U* test. ns, not significant, *P < 0.05, **P < 0.01. **F** Comparison of age-associated B cell (ABC) module scores among B cell subclusters in the discovery cohort. Box: IQR; line: median; whiskers: 1.5 × IQR range; outside points: outliers. Statistical significances were determined using the Mann–Whitney *U* test. ns not significant, **P < 0.01, ***P < 0.001, ****P < 0.0001. **G** Comparison of module scores for isotypes switching and immunoglobulin production gene sets among active B cells. Box: IQR; line: median; whiskers: 1.5 × IQR range; outside points: outliers. Statistical significances were determined using the Mann–Whitney *U* test. ns not significant, *P < 0.05, ***P < 0.001, ****P < 0.0001. **H** Distribution of immunoglobulin isotypes in active B cells from HC, SLE, and LN groups of the discovery cohort. **I** The proportion of active B cells expressing IGHA1 and IGHG1 genes among HC, SLE, and LN groups from the discovery cohort. **J** Somatic hypermutation frequency in each B cell subcluster among HC, SLE, and LN groups from the discovery cohort. Box: IQR; line: median; whiskers: 1.5 × IQR range; outside points: outliers. Statistical significances were determined using the Mann–Whitney *U* test. ns, not significant, *P < 0.05, **P < 0.01, ***P < 0.001, ****P < 0.0001.

B cells play an essential role in the pathogenesis of lupus by producing autoantibodies, presenting antigens, and modulating immune responses, which drive inflammation and tissue damage. Our single-cell analysis identified four distinct B cell subsets: *IGKC*^+^ naive B cells, *IGLC2*^+^ naive B cells, active B cells, and memory B cells (Fig. 2A; Supplementary Fig. 2A). In lupus patients, both *IGKC*^+^ and *IGLC2*^+^ naive B cells showed shorter lengths of heavy chain complementarity-determining region 3 (CDR3) compared to HCs (Fig. 2B; Supplementary Fig. 2B, Supplementary Data 3), corroborating previous studies of CDR3 shortening in autoimmune conditions^21,22^. Principal component analysis (PCA) of V-J gene usage patterns demonstrated that *IGKC*^+^ naive B cells exhibited the most pronounced repertoire differences between lupus patients and HCs, with principal components effectively distinguishing lupus patients from controls (Fig. 2C; Supplementary Fig. 2C). Among V-J genes which highly correlated with principal components, the frequency of *IGHV3-48* and *IGHV3-49* genes were significantly higher in *IGKC*^*+*^ naive B cells of lupus patients (Fig. 2D and E; Supplementary Data 3). These observations are consistent with the data obtained from B cell-enriched PBMC data^15^, indicating a robust pattern that may be characteristic of the disease’s immunological signature (Supplementary Fig. 2D–F). However, the presence of B cell clonal expansion within PBMCs is not apparent, a characteristic that is consistent across both individuals with lupus and those in the HC group (Supplementary Fig. 2G).

Class-switch recombination (CSR) and somatic hypermutation (SHM) were predominantly detected in memory B and active B cells. Active B cells exhibited obvious expression of autoimmune-related age-associated B cell (ABC) signature genes (*ITGAX*, *TBX21*, *ZEB2*)^23–25^, with active B cells from lupus patients showing significantly higher ABC gene module scores compared to HCs (Fig. 2A and F; Supplementary Data 4). Transcriptomic profiling further demonstrated enhanced immunoglobulin production pathways and upregulated regulation of isotype switching to IgG/IgA in lupus-derived active B cells (Fig. 2G; Supplementary Data 3 and 4). Among all B cell subclusters, active B cells exhibited the most pronounced isotype alterations in lupus patients, particularly showing increased *IGHG1* and *IGHA1* proportions in LN compared to SLE patients and HCs (Fig. 2H and I; Supplementary Fig. 2H and I). This implicates a potential contribution of ABC-like active B cells to LN pathogenesis through isotype switching from IgM to IgG1 and IgA1. While SHM—a process critical for B cell affinity maturation that predominantly occurs in germinal centers^26,27^—has shown conflicting reported frequencies in lupus patients^22,28,29^, our data corroborate the observation of reduced SHM frequencies in lupus B cells (Fig. 2J). This finding supports the prevailing concept of enhanced extrafollicular B cell differentiation in the pathogenesis of lupus.

### Enhanced clonal expansion and V(D)J gene preference characterize CD8^+^ effector T cells in LN patients

**Fig. 3:**
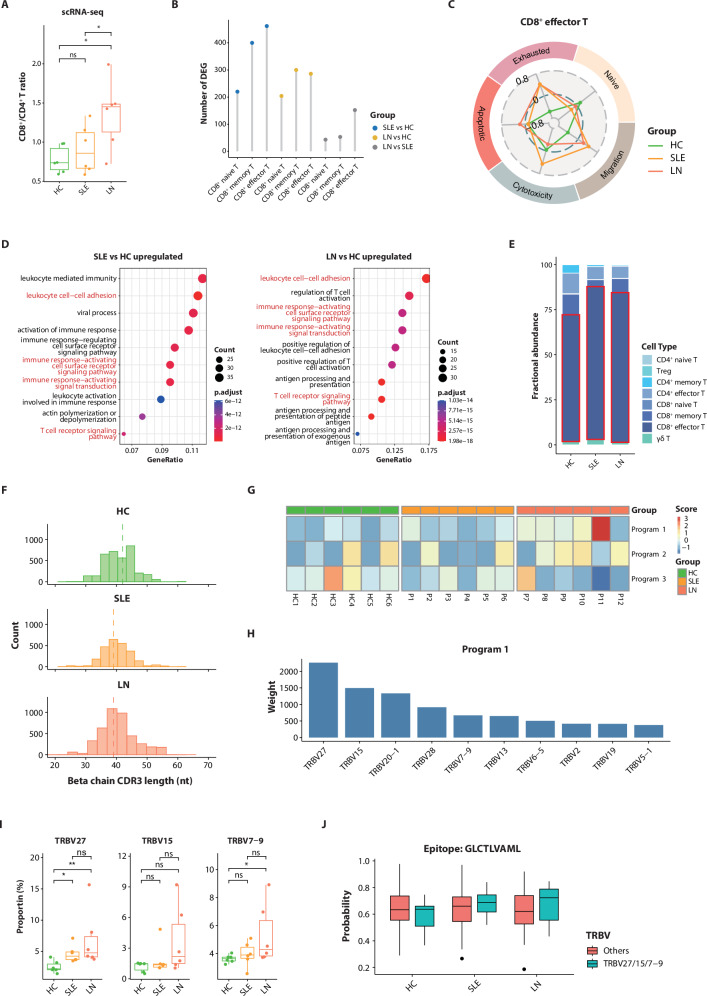
Increasing CD8^+^ T cells and TCR clonal preference were shown in LN patients. **A** Comparison of the CD8^+^ to CD4^+^ T cell ratio among SLE, LN, and HC groups from the discovery cohort. Box: IQR; line: median; whiskers: 1.5 × IQR range from the quartiles; dots: individual samples (*n* = 6 per group). Statistical significances were determined using the Mann–Whitney *U* test. ns not significant, *P < 0.05. **B** Number of differentially expressed genes (DEGs) per CD8^+^ T cell subcluster across group comparisons in the discovery cohort. Data points are color-coded by comparison group. **C** Radar chart shows the average value of scaled module scores for T cell functional gene sets across HC, SLE, and LN groups in the discovery cohort. **D** Top 10 enriched Gene Ontology (GO) terms for genes upregulated in CD8^+^ effector T cells from SLE or LN patients versus HC within the discovery cohort. **E** Stacked bar chart showing the compositional structure of clonally expanded T cells across HC, SLE, and LN groups in the discovery cohort. **F** CDR3 length distribution of the TCR beta chain in CD8^+^ effector T cells across HC, SLE, and LN groups (discovery cohort). **G** Heatmap illustrates the TRBV gene pattern scores in CD8^+^ effector T cells for each individual of the discovery cohort. **H** Top 10 weighted genes in program 1 identified from the discovery cohort. **I** Proportion of LN-preferred TRBV genes across HC, SLE, and LN groups in the discovery cohort. Box: IQR; line: median; whiskers: 1.5 × IQR range from the quartiles; dots: individual samples (*n* = 6 per group). Statistical significances were determined using the Mann–Whitney *U* test. ns not significant, *P < 0.05, **P < 0.01. **J** Binding scores of SLE epitope GLCTLVAML to CD8^+^ effector T cell clones in the discovery cohort were predicted by epiTCR. Predicted binding scores are compared between clones grouped based on LN-associated TRBV gene expression: those expressing these genes versus those not expressing them. Data are presented as a box plot (Box: IQR; line: median; whiskers: 1.5 × IQR range from the quartiles).

T cells in SLE recognize and respond to self-antigens, promoting the development of autoimmune responses and playing a complicated role in immune tolerance and inflammatory processes. For T cell subclusters (Supplementary Fig. 3A), the predominant feature alterations of LN patients are manifested in CD8^+^ T cells. Consistent with prior reports^30,31^, we observed a significantly elevated CD8^+^/CD4^+^ T cell ratio in lupus patients, particularly in those with LN (Fig. 3A; Supplementary Data 5). Among CD8^+^ T cell subclusters, CD8^+^ effector T cells exhibit the most pronounced variability in transcriptome profiles across LN, SLE, and HC (Fig. 3B). This subcluster displayed elevated exhaustion and migration scores in lupus patients compared to HC (Fig. 3C; Supplementary Data 4). Pathway enrichment analysis revealed the activation of cell adhesion, immune response pathways, and TCR signaling in both SLE and LN groups, suggesting shared mechanisms of T cell dysregulation (Fig. 3D).

Despite significant individual variability in the proportion of clonally expanded T cells, clonally expanded CD8^+^ effector T cells were found to be predominant and exhibited a higher prevalence among lupus patients (Fig. 3E; Supplementary Fig. 3B, Supplementary Table 4). TCR repertoire analysis revealed lupus-associated perturbations in CDR3. Specifically, a notable reduction in the length of CDR3 loops within the TCRβ chain was observed in CD8^+^ effector T cells, comparing both SLE and LN groups to HCs (Fig. 3F and Supplementary Fig. 3C). We subsequently examined the TCRβ chain gene usage of CD8^+^ effector T cells and observed obvious alterations in the top 10 TRB variable (TRBV) genes between LN and HCs (Supplementary Fig. 3D).

Non-negative matrix factorization (NMF) decomposition of TRBV gene usage further revealed three distinct programs, with Program 1 showing marked enrichment in LN patients compared to HC (Fig. 3G and Supplementary Fig. 3E and F). The top-weighted TRBV genes in Program 1 (*TRBV27*, *TRBV15*, *TRBV7-9*) corresponded precisely to the most frequently utilized variable genes in LN patients (Fig. 3H and I).

Intriguingly, we identified disease-specific pairing preferences between TRAV and TRBV genes in CD8^+^ effector T cells, with LN patients exhibiting recurrent combinations, including *TRBV27-TRAV14/DV4*, *TRBV15-TRAV17*, and *TRBV7-9-TRAV20* (Supplementary Fig. 3G). Computational prediction using epiTCR^32^ revealed enhanced binding propensity of these clonally expanded TCRs that utilized the *TRBV27/15/7-9* genes to the HLA-A*02:01-restricted Epstein–Barr virus (EBV) epitope GLCTLVAML, a known lupus-related epitope, in lupus patients, particularly in LN patients (Fig. 3J; Supplementary Table 5). This EBV-derived epitope showed significantly stronger predicted binding affinity in lupus patients compared to HCs, suggesting potential involvement of EBV antigen exposure or molecular mimicry mechanisms in driving pathogenic TCR activation during lupus progression.

### Dysregulation of circulating lymphoid cells predicts LN development in SLE

**Fig. 4:**
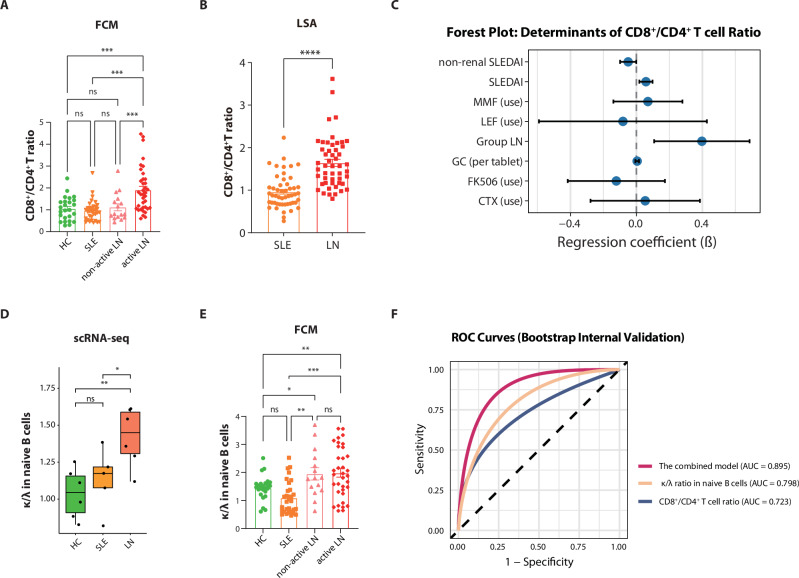
Lymphocyte perturbations as predictive markers for lupus nephritis. **A** The ratio of CD8^+^/CD4^+^ T cells measured by flow cytometry (FCM). Data are presented as mean ± standard error of the mean (SEM). 112 individuals were analyzed, including HCs (*n* = 25), SLE patients (*n* = 34), non-active LN patients (*n* = 16), and active LN patients (*n* = 37). Statistics were assessed by ANOVA and Fisher’s least significant difference test. ns, not significant, ***P < 0.001. **B** Comparison of CD8^+^/CD4^+^ T cell ratio between SLE (*n* = 50) and LN (*n* = 50) was assessed through lymphocyte subset analysis (LSA) from their clinical laboratory tests. Data are presented as mean ± SEM. The statistical significance of the differences was determined using an independent samples *t*-test. ****P < 0.0001. **C** Forest plot from multivariable linear regression analysis evaluating associations between clinical factors and the CD8⁺/CD4⁺ T cell ratio (*n* = 187). The model was adjusted for daily glucocorticoid dose (GC, per tablet), use of mycophenolate mofetil (MMF), leflunomide (LEF), tacrolimus (FK506), cyclophosphamide (CTX), and disease activity (SLEDAI and non-renal SLEDAI scores). Points represent regression coefficients (*β*) with horizontal lines indicating 95% confidence intervals. The vertical dashed line denotes the null effect (*β* = 0). A significantly higher CD8⁺/CD4⁺ ratio was observed in patients with LN (Group LN) compared to those without LN. A positive association was identified between the ratio and the overall SLEDAI score, whereas a negative association was observed with the non-renal SLEDAI scores. No significant associations were found for any immunosuppressive medications. **D** Box plots show the κ to λ light chain ratio in naive B cells from the discovery cohort. Box: IQR; line: median; whiskers: 1.5 × IQR range from the quartiles; dots: individual samples (*n* = 6 per group). One data point was removed from the SLE group as it was determined to be a significant outlier. Statistical significances were determined using the Mann–Whitney *U* test. ns not significant, *P < 0.05, **P < 0.01. **E** The ratio of κ-expressing to λ-expressing naive B cells was measured by FCM. Data are presented as mean ± SEM. 105 individuals were analyzed, including HCs (*n* = 25), SLE patients (*n* = 30), non-active LN patients (*n* = 15), and active LN patients (*n* = 35). Statistics were assessed by ANOVA and Fisher’s least significant difference test. ns, not significant, *P < 0.05, **P < 0.01, ***P < 0.001. **F** Bootstrap-validated ROC curves comparing the combined logistic regression model (AUC = 0.895, 95% CI: 0.820–0.956) with single-predictor models for discriminating LN (*n* = 50) from non-renal SLE (*n* = 30). Individual predictors showed AUCs of 0.798 (95% CI: 0.693–0.892) for κ/λ ratio in naive B cells and 0.723 (95% CI: 0.614–0.826) for CD8⁺ T/CD4⁺ T ratio. Performance was internally validated using 1000 bootstrap resamples. The diagonal dashed line represents chance-level discrimination.

To validate these scRNA-seq findings indicating elevated CD8^+^/CD4^+^ T cell ratios in LN, we performed flow cytometry (FCM). The FCM results confirmed a significant increase in LN patients, particularly those with active nephritis (Fig. 4A; Supplementary Fig. 4A, B, Supplementary Data 6). External validation using an independent scRNA-seq cohort (GSE174188)^33^ further confirmed higher CD8⁺/CD4⁺ T cell ratios in LN patients (Supplementary Fig. 4C). To further substantiate these findings in a clinical context, we assessed clinical lymphocyte subset analysis (LSA) results from 100 lupus patients (Supplementary Data 7). This analysis not only confirmed the elevated CD8⁺/CD4⁺ T cell ratio in LN but also revealed a strong positive correlation of this ratio with global disease activity (SLEDAI: *r* = 0.352, *p* < 0.01) and renal-SLEDAI (rSLEDAI: *r* = 0.47, *p* < 0.01), while no significant correlation was observed with non-renal SLEDAI scores (Fig. 4B; Supplementary Fig. 4D). In multivariable analysis, a diagnosis of LN was strongly and independently associated with an elevated CD8⁺/CD4⁺ T cell ratio, whereas SLEDAI showed only a weak positive association. None of the treatment-related factors reached statistical significance, highlighting that LN itself—rather than overall disease activity or immunosuppressive treatments—is the key clinical factor driving this imbalance in T-cell subsets (Fig. 4C). Absolute cell counts demonstrated CD8^+^ T cell expansion as the primary driver of this imbalance (LN: 738 ± 371 vs. SLE: 429 ± 198 cells/μL; *p* < 0.0001; Supplementary Fig. 4E). These observed changes were accompanied by transcriptomic and TCR alterations in CD8^+^ effector T cells of LN patients, suggesting that CD8^+^ T cells may play an important role in triggering renal flares in LN.

In naive B cells, we observed an elevated κ/λ light chain ratio in LN patients compared to SLE and HCs, with no significant differences detected in memory or active B cell subclusters (Fig. 4D; Supplementary Fig. 4F, Supplementary Data 8). External validation using an independent scRNA-seq cohort (GSE174188) confirmed these findings: By reassigning B cell subtypes using a custom reference dataset defined in this study, naive B cells also revealed a higher κ/λ ratio in the LN group (Supplementary Fig. 4G). We further validated this increase at the protein level via FCM, which demonstrated elevated surface immunoglobulin (sIg) κ/λ ratios on naive B cells from LN patients (Supplementary Fig. 4H and I). Notably, unlike the renal activity-associated CD8⁺/CD4⁺ T cell ratio, the naive B cell κ/λ ratio remained consistent across different renal flare states (Fig. 4E). Multivariable analysis indicated that LN diagnosis was significantly and independently associated with an elevated κ/λ ratio in naive B cells. Other variables—including SLEDAI score, glucocorticoid use, and immunosuppressive treatments—did not show statistically significant associations (Supplementary Fig. 4J). Although the underlying mechanisms still require further investigation, previous studies have suggested that λ-light-chain-positive B cells may mitigate auto-reactivity through the rearrangement of λ chains during receptor editing^34,35^. Our findings indicate that LN patients may exhibit a preference for κ-light-chain expression in naive B cells. This specific alteration could potentially serve as a biomarker for renal involvement in lupus.

Linear regression analysis revealed no significant linear relationship between the κ/λ ratio in naive B cells and the CD8⁺/CD4⁺ T cell ratio, indicating that these two immunologic parameters are independent of each other (Supplementary Fig. 4K). We subsequently integrated these two complementary lymphocyte perturbation biomarkers—the CD8^+^/CD4^+^ T cell ratio and naive B cell κ/λ ratio—into a combined logistic regression model to discriminate LN from non-renal SLE. Constructed using flow cytometry data from a cohort of 80 participants (50 LN and 30 non-renal SLE patients), the model underwent bootstrap internal validation with 1,000 resamples to assess performance and robustness. The combined model achieved an AUC of 0.895 (95% CI: 0.820–0.956), significantly outperforming each individual marker (Fig. 4F). Mechanistically, this model synergistically captures both CD8^+^ T cell-driven renal inflammation and naive B cell tolerance defects. These specific immune signatures demonstrated high value in distinguishing patients with LN from those without (AUC = 0.895), providing a framework for future validation of its utility in stratifying renal risk. Future large-scale, prospective studies are warranted to validate its predictive value for renal outcomes.

### Increased antigen-presenting cell activity and cellular interactions in lupus

**Fig. 5:**
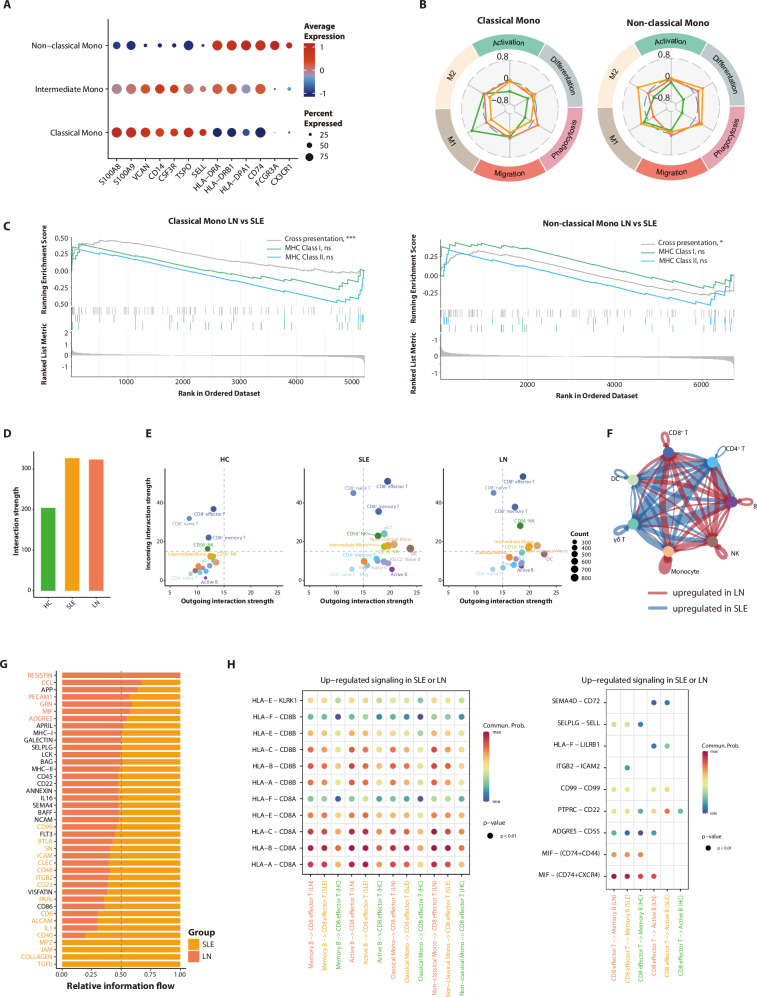
Enhanced antigen presentation and cellular interactions in lupus patients. **A** Dot plot illustrating the expression of marker genes in each monocyte subset. The colors on the plot represent the average expression of the marker genes, while the size of the dots indicates the proportion of cells expressing these genes. **B** Radar charts show the average scaled module scores for functional gene sets in classical and non-classical monocyte subclusters of the discovery cohort, with comparisons made among HC, SLE, and LN groups. **C** Gene set enrichment analysis (GSEA) plots for antigen presentation-related functional gene sets in the discovery cohort. Genes were ranked based on gene expression fold change between the LN and SLE groups in classical and non-classical monocytes. *P* values are estimated by a permutation test. ns, not significant, *P < 0.05, ***P < 0.001. **D** Bar plots show the strength of cellular ligand-receptor interactions in different groups from the discovery cohort. **E** Scatter plots showing the incoming and outgoing strengths for each cell type in each group of the discovery cohort. **F** Differential cell–cell interaction strengths among major immune cell types between LN and SLE groups in the discovery cohort. Line width indicates the magnitude of interaction strength; wider lines represent stronger interactions. Line color denotes the group in which the interaction is upregulated: red for stronger interactions in the LN group, blue for stronger interactions in the SLE group. Labels have been added directly to the figure for clarity. **G** Differential information flow of immune-related signaling pathways in the discovery cohort. Pathways are ranked by their mean differential information flow (LN-SLE). Bar length represents the absolute magnitude of the difference. The color of the pathway labels indicates the group of significant upregulation (FDR < 0.05), as defined by the group’s color. **H** Dot plots display the communication probability in ligand-receptor pairs up-regulated in SLE or LN compared with HC in the discovery cohort. Left panel: Signals received by CD8^+^ effector T cells as receptor targets. Right panel: Signals sent by CD8^+^ effector T cells as ligand sources.

Antigen-presenting cells (APCs), such as monocytes and DCs, play a dual role in lupus pathogenesis by initiating autoimmune cascades while maintaining immunoregulatory potential. Unsupervised clustering resolved monocytes into three distinct subclusters based on marker gene expression patterns (Fig. 5A). Subsequent quantitative and functional analyses revealed lupus-specific monocyte alterations. Compared to HCs, lupus patients exhibited an increased proportion of classical and intermediate monocytes, accompanied by decreased non-classical subsets (Supplementary Fig. 5A). Classical and intermediate monocytes exhibited similar transcriptomic profiles, with lupus patients showing lower scores for classically activated macrophages (M1) differentiation and higher scores for cell activation, differentiation, and phagocytosis. Strikingly, non-classical monocytes demonstrated elevated alternative activated macrophages (M2) differentiation score in both SLE and LN groups (Fig. 5B; Supplementary Fig. 5B, Supplementary Data 4). This aligns with renal biopsy evidence of M2 macrophage predominance in LN^36^, and Arazi et al.’s observation of inflammatory CD16^+^ macrophage transition to M2-like phenotypes in LN kidneys^13^. Our findings suggest that peripheral blood non-classical monocytes from lupus patients are more prone to differentiate towards M2 and may infiltrate into affected organs.

We observed marked upregulation of HLA genes in lupus monocytes, particularly within the non-classical subcluster (Supplementary Fig. 5C). Both MHC class I and II genes were significantly upregulated in lupus monocytes compared to HCs. Notably, MHC-I expression was substantially higher in patients with LN than in those with non-renal SLE (Supplementary Fig. 5D). In contrast, type I and II interferon response genes did not differ significantly between SLE and LN groups, though both exhibited markedly elevated expression compared to HCs (Supplementary Fig. 5E). Subsequent Gene Set Enrichment Analysis (GSEA) confirmed that monocyte subclusters from SLE and LN exhibited upregulation of MHC-I and MHC-II mediated antigen presentation and cross-presentation pathways compared to HCs (Supplementary Fig. 5F and G). Notably, cross-presentation pathway enrichment distinguished LN from SLE in both classical and non-classical monocytes (Fig. 5C).

To systematically characterize intercellular crosstalk alterations, we conducted cell-cell communication analysis using CellChat^37^. Global interaction strength was significantly elevated in lupus patients compared to HCs, consistent with the activation status of individual immune clusters (Fig. 5D). Cytotoxic lineages, including CD8^+^ effector T, γδ T, and CD56^+^ NK cells, displayed enhanced receptor signaling in patients. In contrast, monocyte subclusters, active B cells, and DCs exhibited enhanced outgoing interaction potential, indicating elevated antigen-presenting capacity in these APCs (Fig. 5E). Signaling pathway contribution analysis across SLE, LN, and HC groups identified disease-specific interaction signatures. Both the SLE and LN group showed pronounced enrichment of autoimmune-associated pathways, including BAFF, APRIL, FLT3, and CD48 signaling (Supplementary Fig. 5H), which are established mediators of lupus pathogenesis^38–40^. Direct comparison between SLE and LN revealed amplified interactions among CD8^+^ T cells, monocytes, NK cells, and B cells in LN (Fig. 5F). Information flow quantification demonstrated that LN-specific enhancements predominantly involved pathways related to cell adhesion, migration, and activation (Fig. 5G; Supplementary Fig. 5I).

We identified LN-specific communication hubs by analyzing ligand-receptor pairs in cell types exhibiting both enhanced interaction strength and significant alterations in LN. Focusing on CD8^+^ effector T cells as receptors interacting with B cells/monocytes (ligands), MHC-I-mediated interactions were upregulated in both SLE and LN compared to HCs, with LN showing more pronounced activation. This is consistent with our earlier findings of intensified antigen cross-presentation-driven CD8^+^ T cell responses (Fig. 5H). Strikingly, the MIF-(CD74-CXCR4) ligand–receptor pair displayed stronger activity in LN than in SLE, particularly when memory and active B cells acted as receptors (Fig. 5H). Mechanistically, MIF—a pleiotropic cytokine governing leukocyte chemotaxis—initiates signaling through CD74 co-receptor complexes with CXCR4/CD44^41,42^. Our observation aligns with several clinical studies: First, circulating MIF levels positively correlate with SLEDAI scores in lupus patients^43,44^; second, intrarenal MIF overexpression associates with severe histopathological injury in proliferative glomerulonephritis^45^; third, B cell CXCR4 expression was notably enhanced in lupus patients, especially those with LN or high disease activity^46^. Collectively, our interactome profiling implicates the MIF-(CD74 + CXCR4) axis as a potential driver bridging dysregulated chemotactic signaling to renal leukocyte infiltration in LN.

### Cross-tissue immune profiling reveals that circulating immune cells can partially serve as a reflection of the renal immune landscape in lupus nephritis

**Fig. 6:**
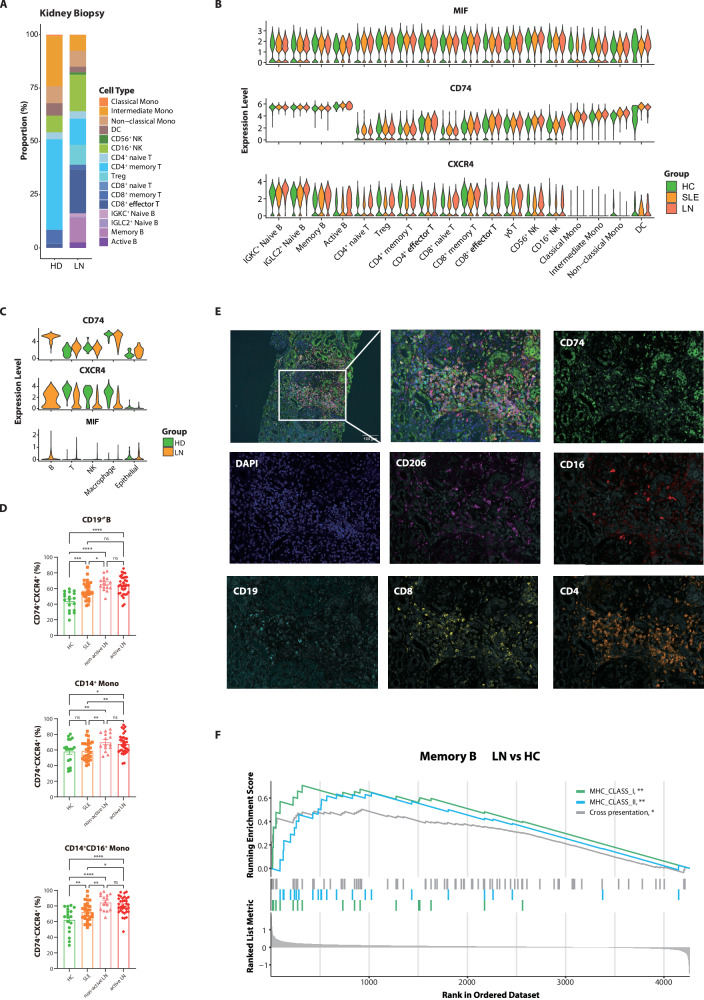
Excessive activation of the macrophage migration inhibitory factor (MIF) pathway in PB and kidney tissues of LN patients. **A** Immune cell composition in kidney biopsies was analyzed from a published scRNA-seq dataset. Bar plot shows the cell proportions calculated from the dataset for lupus nephritis (LN) patients and healthy donors (HD). **B** Expression of the MIF-(CD74 + CXCR4) ligand-receptor pair in circulating immune cells from the discovery cohort. **C** Expression of the MIF receptor complex (CD74 + CXCR4) across major renal cell types. The expression levels are shown for leukocytes and epithelial cells in kidney biopsies from healthy donors (HD) and LN patients (LN). **D** The proportion of CD74^+^CXCR4^+^ in PB CD19^+^ B cells, CD14^+^ monocytes, and CD14^+^CD16^+^ monocytes across HC (*n* = 18), SLE (*n* = 29), non-active LN (*n* = 15), and active LN (*n* = 34) groups. Data are presented as mean ± SEM. Statistics were assessed by ANOVA and Fisher’s least significant difference test. ns, not significant, *P < 0.05, **P < 0.01, ***P < 0.001. **E** mIHC staining for CD4 and CD8 (T cell markers), CD19 (B cell marker), CD16 and CD206 (macrophage markers), CD74 and DAPI. Scale bar represents 100 μm. **F** GSEA plots for antigen presentation-related functional gene sets from the discovery cohort. Genes were ranked based on gene expression fold change of memory B cell subclusters comparing LN to HC groups. *P* values are estimated by a permutation test. *P < 0.05, **P < 0.01.

We cross-referenced our findings with published scRNA-seq data from kidney biopsies^13^, assessing the concordance of immune cell characteristics between peripheral blood and the kidney. Utilizing our data and cell annotations as a reference, we reassigned cell types to immune cells from kidney biopsies, finding that our annotations closely matched the original study (Supplementary Fig. 6A). However, the phenotypes of monocyte-derived macrophages can differ by environment, suggesting our circulating monocyte classifications might not accurately reflect kidney biopsy macrophage subclusters, possibly only indicating their myeloid lineage or transcriptional similarities. Renal immune landscapes in LN biopsies were dominated by lymphoid expansion, reflecting the alterations observed in peripheral blood: clonally expanded CD8^+^ effector T cells, identified in peripheral blood, demonstrated substantial renal infiltration, whereas B cell infiltration was predominantly characterized by *IGKC*^+^ naive, memory, and active B cell subclusters (Fig. 6A; Supplementary Fig. 6B, Supplementary Data 9). The elevated migration scores in peripheral CD8^+^ effector T and B cell clusters (Fig. 3C; Supplementary Fig. 6C) suggest that enhanced migration likely contributes to the accumulation of lymphocytes in the kidney.

Functioning as a ligand for CXCR4, MIF exerts chemokine-mimetic activity to orchestrate the chemotactic recruitment of diverse immune lineages through CD74-dependent mechanisms^47,48^. CellChat analysis revealed enhanced MIF-(CD74 + CXCR4) interactions in LN, paralleled by upregulated *CD74* and *CXCR4* transcripts in peripheral B cells, monocytes, and effector T cells (Fig. 6B; Supplementary Fig. 6D). Immune cells from kidney biopsies exhibited a comparable pattern of CD74 expression (Fig. 6C). While MIF expression remained comparable in circulating immune cells across groups, renal epithelial cells showed elevated *MIF* upregulation in LN biopsies (Fig. 6C). FCM analysis confirmed pan-population CXCR4 protein elevation in lupus, indicating activated circulating immune cells upregulated CXCR4 for migration and organ infiltration in lupus patients. However, in LN patients, elevated CD74 expression was exclusively observed on B cells, classical and intermediate monocytes (Supplementary Fig. 6E). Co-expression analysis further demonstrated LN-specific expansion of CD74^+^CXCR4^+^ dual-positive cells within these subsets (Fig. 6D; Supplementary Fig. 6F). Notably, the elevated CD74 expression did not correlate with disease activity, suggesting it is a common signature of APCs in lupus patients.

Immunohistochemistry (IHC) on LN kidney biopsies localized MIF predominantly to renal tubular epithelial cells, with partial glomerular epithelial expression (Supplementary Fig. 6G), consistent with prior reports^45^. Notably, tubular regions with elevated MIF expression exhibited pronounced peritubular immune cell infiltration, along with characteristic epithelial remodeling featuring cellular atrophy and regenerative architectural changes. Parallel IHC revealed CD74 localization in infiltrating immune cells, tubular epithelium, and glomerular mesangial cells (Supplementary Fig. 6H). To resolve cell-type specificity, multiplex IHC (mIHC) demonstrated CD74 overexpression restricted to CD19^+^ B cells and CD16^+^CD206^+^ M2 macrophages, with minimal expression on CD4^+^ and CD8^+^ T cells (Fig. 6E). Notably, the spatial relationship uncovered that CD74^+^ B cells showed proximity to T cells, especially CD8^+^ T cells. Given CD74’s dual role in chemotaxis and antigen presentation, we further interrogated B cell functionality. Single-cell transcriptomics identified memory B cells as the predominant renal B cell subset, while GSEA of circulating memory B cells revealed LN-specific enrichment of antigen presentation pathways, particularly MHC-I-mediated processes (Fig. 6F).

## Discussion

Peripheral blood is the key route for immune cell transport and a common source for cells that infiltrate diseased tissues. In this study, our systematic immunophenotyping of lupus patients reveals a consistency of nephritis-related immune cell signatures between peripheral blood and renal tissue, establishing circulating biomarkers as non-invasive indicators for monitoring lupus LN progression. We identified two lymphocyte perturbation biomarkers—naive B cell κ/λ light chain imbalance and CD8^+^ T cell predominance—that collectively form a composite model which not only effectively distinguishes patients with LN from those without, but also captures the synergistic pathological mechanisms underlying the disease. Following 1000-replicate bootstrap internal validation, the model demonstrated strong discriminative performance (AUC = 0.895, 80% sensitivity, 83.3% specificity). This approach shows particular promise for clinical settings with limited resources, where renal biopsy is not readily accessible.

Notably, naive B cells exhibited a preference for the κ light-chain in LN patients, regardless of whether they were experiencing renal flares or not. Moreover, this finding is also observable in LN kidney biopsies, where the infiltrated naive B cells are predominantly *IGKC*^+^ naive B cells. It is reported that κ-light-chain-positive B cells are more prone to self-reactivity since λ-light-chain-positive B cells may mitigate auto-reactivity through the rearrangement of λ chains during receptor editing. This aberrant κ light-chain preference in naive B cells might exacerbate autoreactivity and contribute to renal injury. The *IGHV3-48* gene has been recognized as a predictive biomarker for diminished therapeutic response to rituximab in indolent B-cell lymphomas^49^. This finding is consistent with outcomes from the LUNAR study^50^, in which rituximab failed to achieve the anticipated efficacy in LN. In light of our data demonstrating preferential usage of *IGHV3-48* among IGKC⁺ naive B cells in lupus patients, we propose a cross-disease mechanistic hypothesis: *IGHV3-48* may confer intrinsic resistance potential in B cells, possibly through modulation of BCR signaling pathways or alterations in antigen-binding properties. This mechanism may underlie rituximab resistance in lymphomas and attenuated efficacy of B-cell-targeted therapies in lupus. Chimeric antigen receptor (CAR) T-cell therapy targeting B cells is emerging as a promising curative strategy for refractory autoimmune diseases^51^. CD19-directed CAR T cells mediate profound depletion of B-lineage cells, leading to sustained clinical and serological remission in patients with refractory SLE, which persists even after B-cell reconstitution^52,53^. In contrast to rituximab, this approach enables more durable, drug-free disease control, heralding a new era of cellular therapy for autoimmune disorders.

Prior studies have described a trimodal distribution of the peripheral blood CD8⁺/CD4⁺ T cell ratio in SLE, linking a high ratio to renal and hematological involvement, and a low ratio to neurological and musculoskeletal involvement^54^. Additionally, LN patients were reported to have a higher CD8⁺/CD4⁺ T cell ratio than both non-LN patients and healthy controls^55,56^. Our multi-omics analysis revealed that the significantly elevated CD8⁺/CD4⁺ T cell ratio in LN patients correlated with higher rSLEDAI scores and was accompanied by significant clonal expansion of CD8⁺ effector T cells. These CD8^+^ effector T cells exhibit evidence of heightened antigenic stimulation, suggesting that this activation drives their clonal expansion in circulation and facilitates their recruitment to inflamed kidneys. This is consistent with a recent study using scRNA-seq analysis in LN kidney biopsies, which identified GZMK^+^ CD8^+^ T cells as the predominant kidney-associated T cells^14^. The CD8^+^ effector T cells from LN patients exhibit a preferential usage of certain TRBV genes, specifically *TRBV27*, *TRBV15*, and *TRBV7-9*, which have a higher binding affinity for the EBV epitope GLCTLVAML. Furthermore, the genes associated with antigen cross-presentation were significantly enriched in monocytes from lupus patients, with LN patients showing a higher enrichment than SLE patients. Antigen cross-presentation happens when APCs present exogenous antigens on MHC-I molecules, leading to the activation of CD8^+^ T cell responses, which are crucial for antiviral and antitumor immunity, as well as the induction of peripheral tolerance^57,58^. Consequently, we hypothesize that the EBV infection, molecular mimicry, and enhanced antigen cross-presentation in LN patients may induce the clonal expansion and activation of CD8^+^ effector T cells.

Both type I and type II interferons are well-established inducers of MHC-I expression^59–62^. To investigate whether the pronounced MHC-I upregulation observed in monocyte subsets from LN patients is driven by a specific interferon subtype, we analyzed corresponding ISG signatures. We found that both SLE and LN patients exhibited significantly elevated type I and type II interferon responses compared to HCs. However, despite a subtle statistical difference detectable through the high resolution of our single-cell data, the mean interferon scores between SLE and LN groups were nearly identical. Thus, the pronounced MHC-I upregulation in LN is unlikely to be primarily driven by a differential interferon response.

The MIF-(CD74 + CXCR4) pathway is one of the most significantly upregulated ligand-receptor pairs in LN patients. In our analysis, renal epithelial cells from LN patients demonstrated elevated *MIF* expression levels. This is consistent with earlier research on proliferative glomerulonephritis, which reported increased renal MIF levels associated with leukocyte infiltration, tissue damage, and a decline in renal function, suggesting MIF plays a key role in kidney disease progression^45^. CD74, widely expressed on peripheral and renal B cells and monocytes/macrophages, facilitated microenvironmental crosstalk—mIHC revealed spatial colocalization of CD74^+^ B cells with CD8^+^ T cells in renal niches. CD74 is a critical mediator of both MHC class I and II antigen presentation^63^. The memory B cells, the dominant renal B subset, exhibited LN-specific enrichment of MHC-I antigen presentation pathways, suggesting CD74-mediated localized CD8^+^ T cell activation. Therapeutic relevance is highlighted by milatuzumab (humanized anti-CD74 antibody), which modulated B cell behavior and showed efficacy in refractory SLE despite early trial termination (NCT01845740)^64,65^. Based on these results, our study further supports CD74 as a promising therapeutic target for lupus patients, especially for those with nephritis.

Our study has several limitations. First, the absence of renal biopsies in “non-LN” SLE patients precludes definitive exclusion of subclinical nephritis. However, we rigorously selected SLE controls without urinary abnormalities, renal history, and with more than 12-month follow-up, confirming stable renal status. Second, we acknowledge the limited sample size of our original discovery cohort. To address this constraint, key findings were consistently validated across two external scRNA-seq datasets, an internal clinical cohort, and an independent flow cytometry cohort. This multi-tiered validation approach effectively mitigates concerns related to the initial sample size and substantially strengthens the robustness of our conclusions. Third, considering that SLE is a systemic condition, patients may experience damage to organs beyond the kidneys. Consequently, we have strictly included only those patients who do not exhibit significant organ damage apart from renal involvement. Fourth, while peripheral T/B cell clonal biases align with published renal data, unpaired kidney immune repertoire analyses limit direct clonal validation. Finally, the mechanism underlying the kappa light-chain preference phenomenon in B cells remains unclear, and relevant research in this field is notably scarce. Therefore, further comprehensive investigations are necessary to validate these findings.

## Methods

### Study subjects

Ethical approval was obtained from the Ethics Committee of the Chinese People’s Liberation Army General Hospital (Approval Nos. S2019-095-01 and S2022-640-01), and all procedures conformed to the ethical standards set forth in the Declaration of Helsinki. All ethical regulations relevant to human research participants were followed.

For scRNA-seq in our discovery cohort, we recruited 12 SLE patients who fulfilled the 1997 ACR revised classification criteria^66^. Among them, six had active LN, confirmed by kidney biopsy at the time of blood sampling, while the remaining six showed no clinical evidence of kidney injury throughout their disease course. Two independent renal pathologists assessed the ISN-RPS histopathological class^67^ for the six LN patients. None of the participants had received biologic agents or had comorbid autoimmune and inflammatory disorders, and all were age- and sex-matched. To minimize confounding from extra-renal disease activity, patients with active infections or significant non-renal manifestations were excluded. The SLE and LN cohorts were also matched for non-renal SLEDAI scores, ensuring that differences in overall disease activity primarily reflected renal involvement. For the HC group, we applied the same sequencing strategy to peripheral blood from two newly recruited healthy donors and included publicly available data from four additional age- and sex-matched HCs^68,69^.

To validate scRNA-seq findings, we integrated two external datasets. The first was a 5’-end scRNA-seq dataset (GSE193867) comprising CD19⁺ B cell-enriched PBMC samples from three lupus patients and three HCs. These data were merged with our in-house dataset to improve B-cell annotation. The second was a large-scale 3’-end scRNA-seq dataset (GSE174188) including 99 HCs, 68 SLE, and 76 LN samples. Due to substantial technical differences between 3’ and 5’ sequencing protocols, this dataset was used as an independent cohort for validating key results.

For the clinical validation cohort, we included an additional 187 lupus patients and 25 HCs. Flow cytometric (FCM) validation was performed on PBMCs from a subset of 87 patients and all 25 HCs. Additionally, clinical lymphocyte subset analysis (LSA) data were available for a separate group of 100 patients. The SLE group consisted of patients without clinical signs of kidney injury throughout their disease course. The active LN group included patients with physician-diagnosed LN and a renal SLEDAI score of ≥4 at sampling. The non-active LN group comprised patients with a prior LN diagnosis but a renal SLEDAI score of 0 at the time of sampling.

All patients were hospitalized, and their clinical information, including age, gender, SLEDAI components^70^, medication use, and results of clinical laboratory tests, is documented in Supplementary Tables 1 and 2 (scRNA-seq cohort) and Supplementary Data 6 and 7 (clinical validation cohort).

### Processing of blood samples

Peripheral blood was processed within 2 h of collection. After dilution with DPBS, the samples were carefully layered onto an equal volume of Ficoll-Paque PLUS (17144003, Cytiva, Sweden) according to the manufacturer’s instructions. The buffy coat was then aspirated and transferred to a new tube, followed by two washes with DPBS. In cases of residual erythrocyte contamination, cells were incubated with Red Blood Cell Lysis Buffer for 5 min before a final wash. The isolated PBMCs were counted and assessed for viability, which exceeded 85% in all samples. For scRNA-seq, fresh PBMCs were immediately used for library preparation. For flow cytometry, PBMCs were resuspended in SuperKine Serum/Protein-Free Cell Freezing Medium (BMU108-CN, Abbkine, China), cryopreserved at −80 °C for 24 h, and subsequently transferred to liquid nitrogen for long-term storage.

### 10X Genomics scRNA-seq library preparation and sequencing

Freshly isolated PBMCs (viability > 85%) were subjected to scRNA-seq library preparation. Briefly, single-cell suspensions were loaded onto the 10X Genomics Chromium platform to generate barcoded gel bead-in-emulsions (GEMs) using the Chromium Next GEM Single Cell 5’ Kit v2 (PN-1000263) according to the manufacturer’s protocol. Approximately 6000 cells per sample were targeted for capture. Reverse transcription was performed on barcoding RNA, followed by cDNA amplification and quality assessment. Final libraries—incorporating V(D)J enrichment for immune receptor profiling—were constructed and sequenced on an Illumina NovaSeq system at Annoroad Gene Technology (Beijing, China).

### scRNA-seq data processing and cell type annotation

Single-cell sequencing data were aligned and quantified using Cell Ranger (v.6.1.2) with the GRCh38 human reference genome to generate a count matrix for the following analysis. The quality control, batch effect correction, clustering analysis, and UMAP embedding were performed by scanpy (v.1.9.3). Quality control was applied to cells based on UMI count, number of genes, mitochondrial, and globin gene counts. Cells with more than 10% mitochondrial gene or 10% globin gene counts were filtered. For each sample, we calculated the median and median absolute deviation (MAD) for UMI count and number of genes, and removed outlier cells based on the median and three times MAD of UMI counts and number of genes. Then we remove cells with UMI counts above 20,000 or <1000, and detected genes above 5000 or <500. For potential doublets, we applied scrublet^71^ (v.0.2.3) with default parameters to identify and remove doublet cells.

The count data were normalized using *scanpy.pp.normalize_total*, and a logarithmic transformation was performed for the following analysis.

We identified the top 2000 variable genes in PBMC and B-cell data using ‘Seurat_v3’ parameters in scanpy, and applied their union for subsequent principal component analysis (PCA). For PCA, we regressed out the cellcycle scores, S scores, and G2M scores, which are calculated by using the score_genes_cell_cycle. The batch effect correction was performed by Harmony and BBKNN. Then we performed unsupervised clustering and UMAP embedding to identify and visualize cell clusters. The cell types of clusters were assigned according to the expression of marker genes.

### Principle component analysis for cell type proportions

To illustrate the general preferences among the LN, SLE, and HC groups, we conducted a principal component analysis on the cellular composition of the major cell types. We utilized the pcaCoDa function in the R package “robCompositions” to compute the principal components.

### Mutation frequency for SLE-related loci

To ascertain the variants associated with SLE-related genetic locations, we utilized VarTrix, a tool tailor-made for the analysis of single-cell genotyping datasets. For published SLE-related loci, we computed the mutation frequencies for the LN, SLE, and HC groups. Subsequently, we graphically represented the findings using bar plots for a clear visual comparison.

### TCR and BCR analysis

We first assembled and quantified TCR and BCR sequences using the Cell Ranger (v.6.1.2) vdj protocol, referencing the GRCh38 human genome. To improve the precision of TCR and BCR repertoires, we adhered to the dandelion guideline for re-annotation and analysis of antigen receptor sequences. Subsequently, we removed contigs labeled as non-productive or with fewer than two UMIs, retaining only those cells identified as T or B cells within the transcriptomic data.

For clone identification, we employed the find_clones function from the dandelion Python package, which implements a standardized workflow for clustering antigen receptor sequences into clones based on their structural and sequence features. For TCR clones, the algorithm grouped cells into the same clone if they shared identical α and β chain sequences, defined by matching V, D (for β chain), J gene segment usage, and identical CDR3 nucleotide sequences. For BCR clones, the function applied similar core criteria—identical heavy and light chain V(D)J gene combinations and CDR3 nucleotide sequences—while incorporating specific handling of somatic hypermutations typical in B cells. To ensure the analysis focused on clonotypes with the potential to encode functional immune receptors, we filtered out all non-productive sequences. For each sample, expanded clones were defined as those appearing in more than three cells.

For TCR data, we calculated the length of CDR3 and TRBV gene frequencies for CD8^+^ effect T cells in LN, SLE, and HC groups. To identify TRBV gene usage pattern in lupus patients, we performed nonnegative matrix factorization analysis with R package NMF and visualized the program scores by using the pheatmap function.

For BCR data, we calculated somatic hypermutation frequency by the dandelion function *dandelion.pp.quantify_mutations*. For gene usage in IGKC^+^ Naive B cells, we performed principal component analysis using the *dandelion.tl.vj_usage_pca* function.

### Differential expression and functional analysis

Differentially expressed genes (DEGs) were identified through the FindMarkers or FindAllMarkers function in the Seurat package (v4.3.0) and utilizing the MAST (v1.8.2) method. Thresholds of min.pct and fold change were set to 0.3 and 1.5, respectively. Genes with an adjusted *P* value of <0.05 were reserved as DEGs for further analysis.

The “clusterProfiler” R package (v4.4.4) and metascape were used to perform GSEA and enrichment analysis for DEGs. For the functional gene set, we calculate module scores for corresponding cell types by using the *scanpy.tl.score_genes* function.

### Cell–cell communication analysis by CellChat

R package “CellChat” (v1.6.1) was used to assess the cell–cell interactions. Genes expressed in more than 10% of the cells in one cluster, and the ligand–receptor pairs with a *P* value <0.05 were considered as significant interaction molecules between different cell types.

### Validation using published scRNA-seq datasets

To independently validate the key findings of our study, we utilized two external single-cell RNA sequencing datasets from published studies^13,33^. Raw count matrices and original cell type annotations from these external datasets were obtained. We first harmonized gene symbols across all datasets and performed label transfer using the *Seurat* package. This approach effectively re-annotated the cells in the external datasets using the well-defined cell type labels established in our study. Following label transfer results, key disease-relevant statistics, naive B κ/λ light chain ratio, and cell type composition were computed within the re-annotated external datasets. We also evaluated the expression levels of critical gene signatures identified in our study across the transferred cell types in the external cohorts.

### Flow cytometry analysis

Frozen PBMC samples were rapidly thawed at 37 °C and centrifuged in RPMI-1640 medium to remove dead cells before further processing. After incubating with FcR blocking reagent (130-059-901, Miltenyi Biotec, Germany) for 10 min at 4 °C, PBMCs were stained in staining buffer with antibodies (clone numbers in parentheses) specific for the following antigens: CD3 (OKT3), CD4 (RPA-T4), CD8 (SK1), CD19 (HIB19), CD16 (3G8), CD14 (M5E2), CD27 (M-T271), CD74 (LN2), IgM (MHM-88), CXCR4 (12G5), purchased from BioLegend. Anti-Human Kappa Light Chains+ Lambda Light Chains reagent for κ: λ staining of B subpopulations was purchased from DAKO (FR481, Denmark). 7-AAD was used to exclude dead cells. Stained cells were analyzed on a Guava easyCyte system (Luminex, USA). Data were analyzed using FlowJo v10.8.1 software (BD Life Sciences, USA). In all analyses, cells were initially gated as singlets, then as live cells, and subsequently as lymphocytes or monocytes. Gating strategy for the analysis of the κ/λ ratio of B subpopulations included naive B cells (CD3^−^CD19^+^CD27^−^IgM^+^), switched memory B cells (CD3^−^CD19^+^CD27^+^IgM^−^), unswitched memory B cells (CD3^−^CD19^+^CD27^+^IgM^+^), and double-negative B cells (CD3^−^CD19^+^CD27^−^IgM^−^). Gating strategy for the analysis of CD74 and CXCR4 expression in PBMC included CD4^+^ T cells (CD3^+^CD19^−^CD4^+^CD8^−^), CD8^+^ T cells (CD3^+^CD19^−^CD4^−^CD8^+^), CD16^+^ NK cells (CD3^−^CD19^−^CD4^−^CD8^−^CD16^+^), B cells (CD3^−^CD19^+^), classical monocytes (CD14^++^CD16^−^), intermediate monocytes (CD14^+^CD16^+^), and non-classical monocytes (CD14^−^CD16^++^).

### Immunohistochemistry staining of kidney biopsy

Kidney biopsy samples from LN patients are fixed with 4% paraformaldehyde and made into paraffin sections. Immunohistochemical staining was performed on 3 μm-thick consecutive paraffin sections using the primary antibodies against MIF (ab65869, Abcam; 1 μg/ml) with heat-induced antigen retrieval in citrate buffer (pH 6.0), or CD74 (ab270265, Abcam, 1 μg/ml) with heat-induced antigen retrieval in Tris/EDTA buffer (pH 9.0). DAB substrate kit (BD Biosciences, USA) was then used for immunohistochemical staining, according to its protocol. Renal sections were then imaged with a microscope (Leica, Germany).

### Multiplex immunohistochemistry staining

Multiplex immunohistochemistry (mIHC) was performed on a 3 μm-thick paraffin section. The primary antibodies used were rabbit monoclonal (ab134114, Abcam; 1:500 dilution; pH 9.0 retrieval) to CD19, and rabbit monoclonal (ab270265, Abcam; 1 μg/ml; pH 9.0 retrieval) to CD74, and rabbit monoclonal (ab237709, Abcam; 1:2000 dilution; pH 9.0 retrieval) to CD8, and rabbit recombinant multiclonal (ab288724, Abcam; 1:1000 dilution; pH 9.0 retrieval) to CD4, and rabbit monoclonal (ab246222, Abcam; 1:500 dilution; pH 9.0 retrieval) to CD16, and rabbit polyclonal (ab64693, Abcam; 0.1 μg/ml; pH 6.0 retrieval) to CD206. In brief, all slides were deparaffinized, heat-induced epitope retrieval, inactivated with endogenous peroxidase, incubated with primary antibody, incubated with HRP secondary antibody, and labeled with TSA dyes. Immunofluorescent signal was visualized using the OPAL™ 7-color manual IHC kit (Akoya Bioscience), TSA dyes 480, 520, 570, 620, 690, and 780, and counterstained with Spectral DAPI. All slides were imaged on the Vectra Polaris Automated Quantitative Pathology Imaging System (Akoya Biosciences) at ×20 magnification. Color separation and Cell Phenotyping were performed on InForm image analysis software (Akoya Bioscience) to extract image data. Slides were examined for the presence of CD74+ infiltrating immune cells within the kidney biopsy from LN patients.

### Statistics and reproducibility

All statistical analyses were performed using R (v4.0.5) and GraphPad Prism 9. Data handling and visualization were conducted using the tidyverse suite of packages. Group comparisons were made using Student’s *t*-test, Mann–Whitney *U* test, or one-way ANOVA, as appropriate. Associations between continuous variables were assessed by Pearson’s correlation. Multiple linear regression was employed to evaluate the relationships between clinical factors (including SLEDAI, immunosuppressant use, and disease group) and immunologic markers (CD8⁺/CD4⁺ T cell ratio and naive B cell κ/λ ratio). The association between these two immunologic markers was further assessed by simple linear regression. To discriminate between SLE and LN patients, a binary logistic regression model incorporating both the naive B cell κ/λ ratio and the CD8⁺/CD4⁺ T cell ratio was developed. The model’s performance was internally validated via 1000 bootstrap replicates and is reported as the area under the receiver operating characteristic curve (AUC) with a 95% confidence interval (CI). The optimal classification threshold was determined using Youden’s index. A two-sided *P*-value < 0.05 was considered statistically significant. Detailed statistical results for specific comparisons are provided in the respective figure legends.

### Reporting summary

Further information on research design is available in the Nature Portfolio Reporting Summary linked to this article.

## Supplementary information

Supplementary Information

Description of Additional Supplementary files

Supplementary Data 1-9

Reporting Summary

## Data Availability

Numerical source data for all graphs in this manuscript are available in the Supplementary Data files (Supplementary Data 1–9). The sequencing data have been deposited in the Genome Sequence Archive^72^ in the National Genomics Data Center^73^, China National Center for Bioinformation/Beijing Institute of Genomics, Chinese Academy of Sciences (GSA-Human: HRA008899), which are publicly accessible at https://ngdc.cncb.ac.cn/gsa-human.

## Code availability

The codes for the data analysis in this study are available from the corresponding author upon reasonable request.
