# Supplementary material for: Single-cell profiling unveils nephritis-related circulating immunological signatures in systemic lupus erythematosus patients
Source: Commun Biol. 2026 Jan 5;9:155. doi: 10.1038/s42003-025-09431-8 (PMC12868680; doi:10.1038/s42003-025-09431-8)
Supplement: Supplementary file 1 — Supplementary Information [file 42003_2025_9431_MOESM1_ESM.pdf]

Supplementary Information

Single-Cell Profiling Unveils Nephritis-Related Circulating Immunological Signatures in Systemic Lupus Erythematosus Patients

Liu *et al.*

Supplementary materials in the PDF contains

Supplementary Figure 1–6

Supplementary Table 1–5

Other Supplementary Material for this manuscript includes the following:

Supplementary Data 1–9

Supplementary Figures

Supplementary Figure 1. Quality control and an overview of circulating immune cell scRNA-seq data.

Supplementary Figure 2. The BCR variants in lupus patients.

Supplementary Figure 3. The TCR variants in lupus patients.

Supplementary Figure 4. The elevated $CD8^+/CD4^+$ T cell ratio and naive B cell κ/λ ratio in LN group.

Supplementary Figure 5. Supplementary results for monocyte subclusters and cellular interactions.

Supplementary Figure 6. The expression of MIF-(CD74+CXCR4) axis in circulating immune cells and renal cells.

Supplementary Tables

Supplementary Table 1: Overview of scRNA-seq cohort

Supplementary Table 2: Detailed clinical characteristics of the PBMC scRNA-seq discovery cohort

Supplementary Table 3: CellType Count By Patient

Supplementary Table 4: T ExpandedClone CellTypeCount

Supplementary Table 5: Top 10 $CD8^+$ Effector T Cell Clones with Highest Predicted Affinity for GLCTLVAML

Supplementary Data

Supplementary Data 1: Defining marker genes for all identified cell clusters.

Supplementary Data 2: Source data behind Figure 1C.

Supplementary Data 3: Source data behind Figure 2B, 2E, 2G.

Supplementary Data 4: Gene sets used for single-cell module scoring.

Supplementary Data 5: Source data behind Figure 3A.

Supplementary Data 6: Clinical characteristics of the FCM validation cohort and corresponding results (source data for Fig. 4A, C, E, 6D).

Supplementary Data 7: Clinical characteristics of the LSA validation cohort.

Supplementary Data 8: Description: Source data behind Figure 4D.

Supplementary Data 9: Source data behind Figure 6A.

Supplementary Figure 1

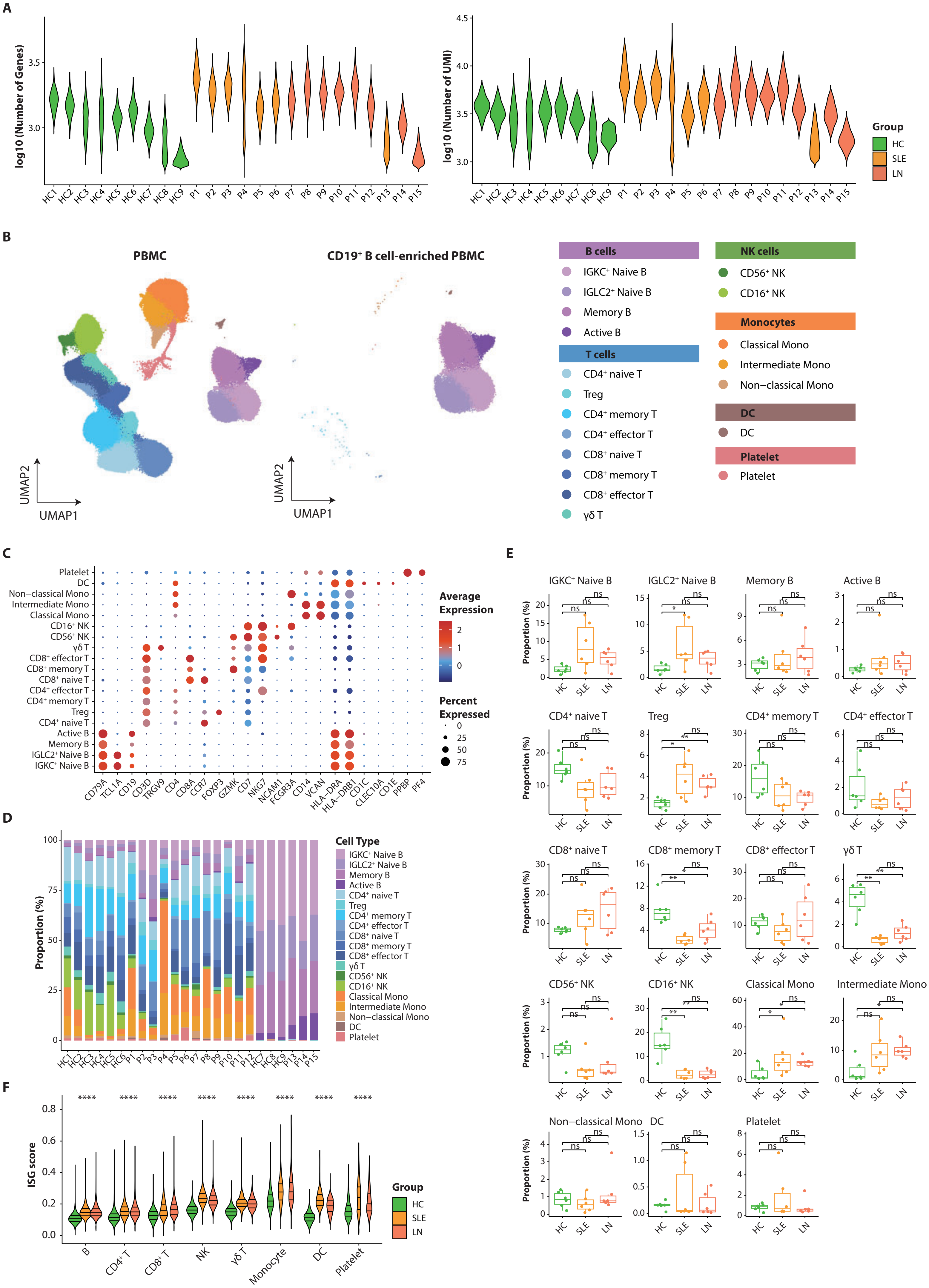

Supplementary Figure 1. Quality control and an overview of circulating immune cell scRNA-seq data.

A. Violin plots show the number of genes (left) and unique molecular identifiers (UMIs, right) per sample. Samples HC1–HC6 and P1–P12 show PBMC scRNA-seq data from the discovery cohort; HC7–HC9 and P13–P15 are from the $CD19^+$ B cell-enriched PBMC scRNA-seq dataset (GEO: GSE193867). Detailed sample information is provided in Supplementary Table 1.

B. Uniform manifold approximation and projection (UMAP) of the integrated 5' scRNA-seq dataset, combining the discovery cohort with the external 5' validation cohort GSE193867. Left: PBMC data from the discovery cohort (n = 6 per group: SLE, LN, HC). Right: $CD19^+$ B cell-enriched data from the GEO dataset GSE193867. Cell numbers per sample are detailed in Supplementary Table 3.

C. Dot plot illustrating the expression of marker genes in each cell subset of the integrated 5' scRNA-seq dataset, which combines the discovery cohort with the external 5' validation cohort GSE193867. Dot color represents average expression; dot size indicates the percentage of expressing cells.

D. Bar plots show the composition of each cell subset in each individual of the integrated 5' scRNA-seq dataset.

E. Box plots illustrate proportion of each cell subset among SLE, LN and HC groups in the discovery cohort. Box: IQR; line: median; whiskers: 1.5×IQR range from the quartiles; dots: individual samples (n = 6 per group). Statistics were assessed by ANOVA and Fisher's least significant difference test. ns, not significant, $*P < 0.05$, $**P < 0.01$.

F. Violin plots show the module scores of interferon-stimulated gene (ISG) in each major cell type between SLE, LN and HC groups in the discovery cohort. Statistical significance of the differences was determined using the Mann-Whitney U test. $****P < 0.0001$.

Supplementary Figure 2

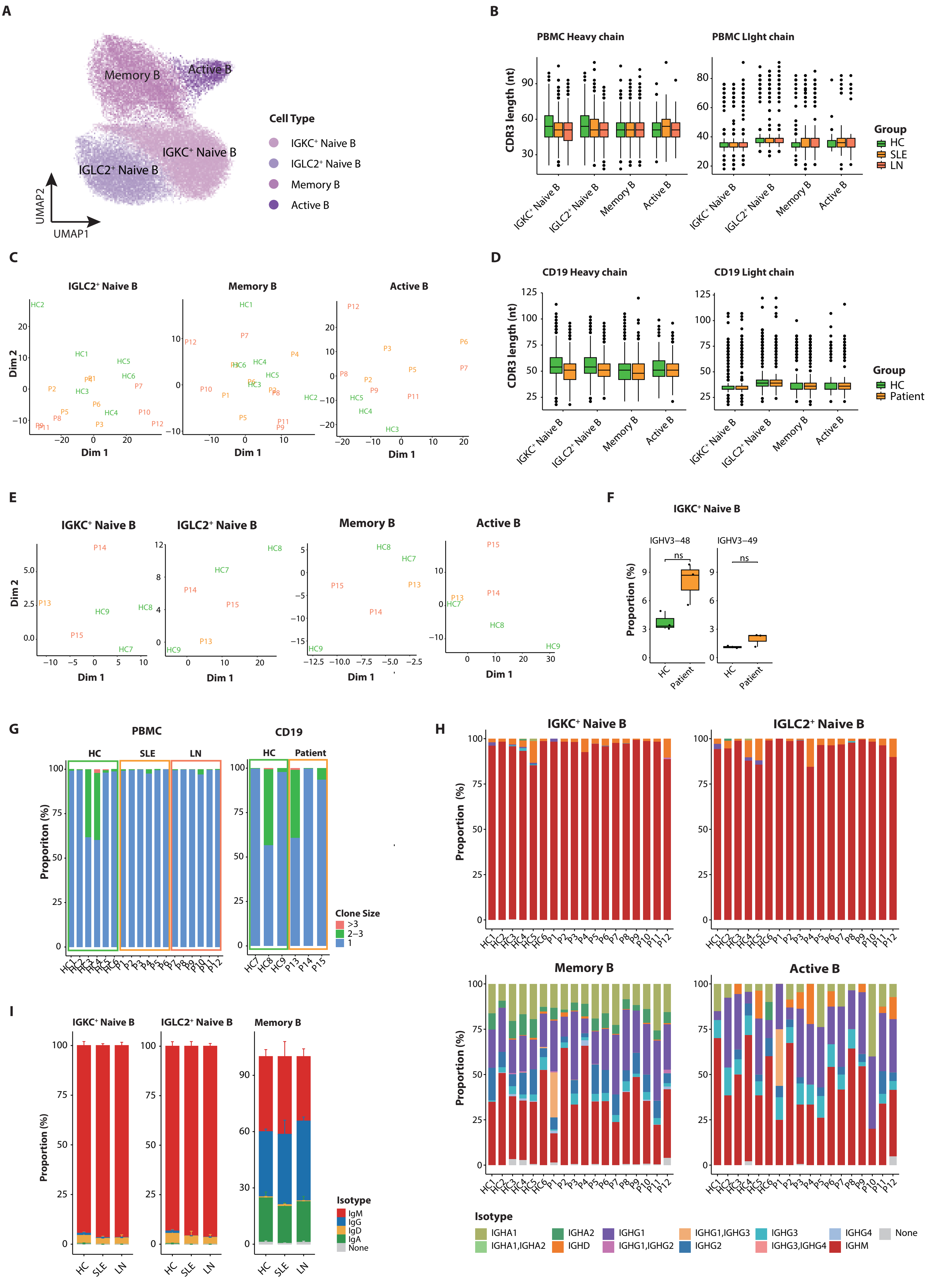

Supplementary Figure 2. The BCR variants in lupus patients.

A. UMAP visualization of the four B cell subclusters.

B. CDR3 length analysis of heavy and light chains across B cell subcluster in the HC, SLE, and LN groups of the discovery cohort. Box: IQR; line: median; whiskers: 1.5×IQR range; outside points: outliers.

C. Principle component analysis of V/J gene usages in IGLC2+ naive, memory and active B cell subclusters from the discovery cohort (n = 6 per group).

D. CDR3 length analysis of the heavy and light chains in B cell subclusters between HC and lupus patients, using B cell-enriched PBMC scRNA-seq data (GSE193867). Box: IQR; line: median; whiskers: 1.5×IQR range; outside points: outliers.

E. Principle component analysis of V/J gene usages in each B cell subset from individuals, based on B cell-enriched PBMC scRNA-seq data (GSE193867).

F. The proportion of IGKC^{+} naive B cells carrying reported heavy chain V genes in lupus patients versus HCs, derived from B cell-enriched PBMC scRNA-seq data (GSE193867). Box: IQR; line: median; whiskers: 1.5×IQR range from the quartiles; dots: individual samples (n = 3 per group). Statistical significances were determined using the Mann-Whitney U test. ns, not significant.

G. Stacked bar plots show the proportional composition of clonally expanded B cells per individual in PBMC scRNA-seq data from discovery cohort (left) and B cell-enriched PBMC scRNA-seq data from GSE193867 (right).

H. The proportion of each isotype per B cell subcluster in individuals from the discovery cohort.

I. Isotypes distribution in IGKC^{+} and IGLC2^{+} naïve and memory B cells across HC, SLE and LN groups of the discovery cohort.

Supplementary Figure 3

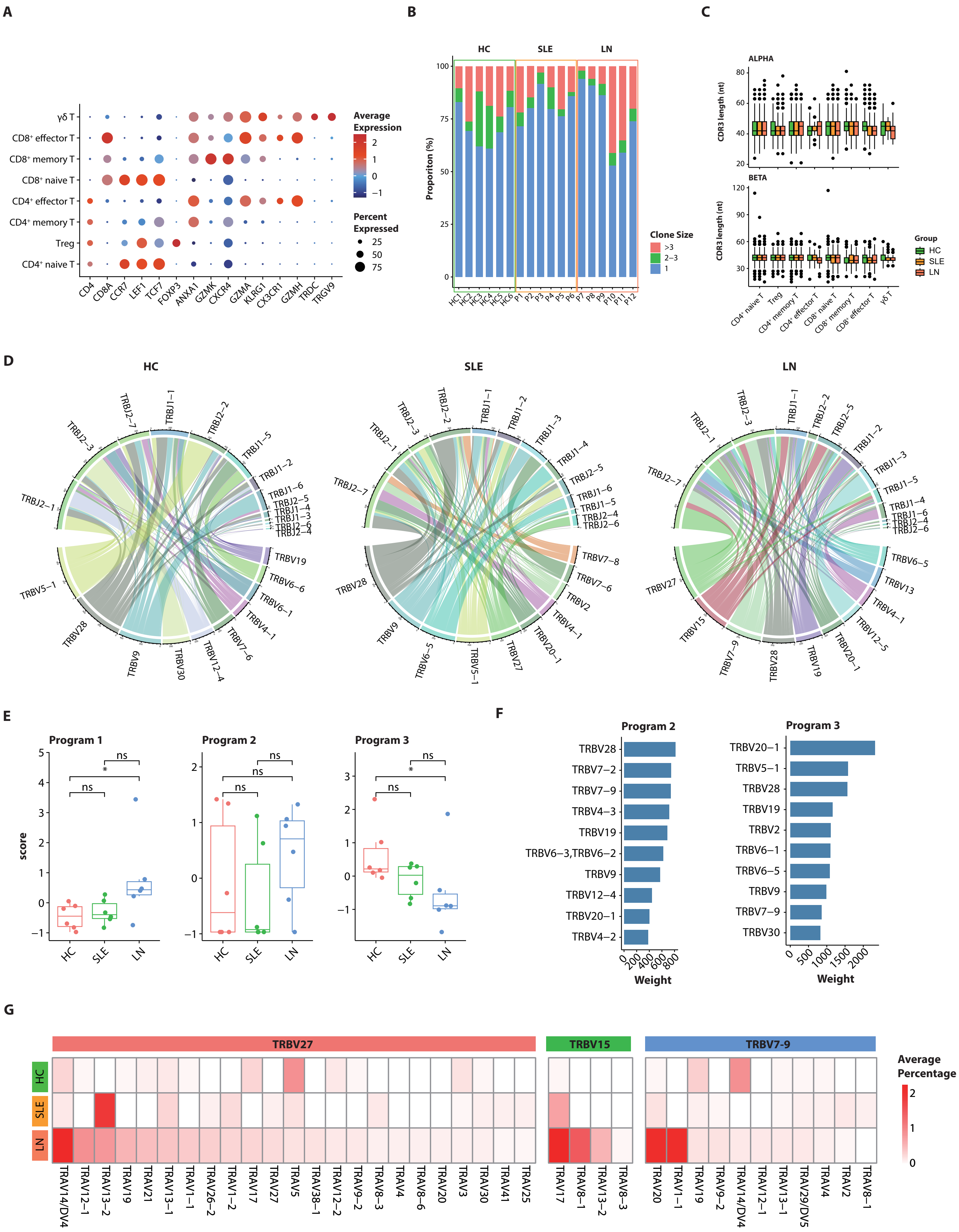

Supplementary Figure 3. The TCR variants in lupus patients.

A. Dot plot illustrating the expression of marker genes in each T cell subset. The colors on the plot represent the average expression of the marker genes, while the size of the dots indicates the proportion of cells expressing these genes.

B. Bar plots show the composition of clonally expanded T cells in each individual from the discovery cohort.

C. CDR3 length of the alpha and beta chain for each T cell subset among SLE, LN and HC groups in the discovery cohort. Box: IQR; line: median; whiskers: 1.5×IQR range; outside points: outliers.

D. Chord graphs display the top 10 V-J gene pairs of beta chain of $CD8^+$ effector T cells from HC, SLE and LN groups in the discovery cohort. Each node around the circle represents a distinct TRBV/J gene, and the chords connecting the nodes indicate the co-occurrence of specific TRBV-TRBJ gene pair. The width of the chords is proportional to the frequency of co-occurrence.

E. Box plots illustrate the scores of each program derived from non-negative matrix factorization (NMF) analysis among SLE, LN and HC groups from the discovery cohort. Box: IQR; line: median; whiskers: 1.5×IQR range from the quartiles; dots: individual samples ($n = 6$ per group). Statistical significances were determined using the Mann-Whitney U test. ns, not significant, $*P < 0.05$.

F. Top 10 weighted genes in program 2 and 3 from the discovery cohort.

G. Heatmap illustrates the average percentage of TRBV-TRAV pairs preferred in SLE and LN patients from the discovery cohort.

Supplementary Figure 4

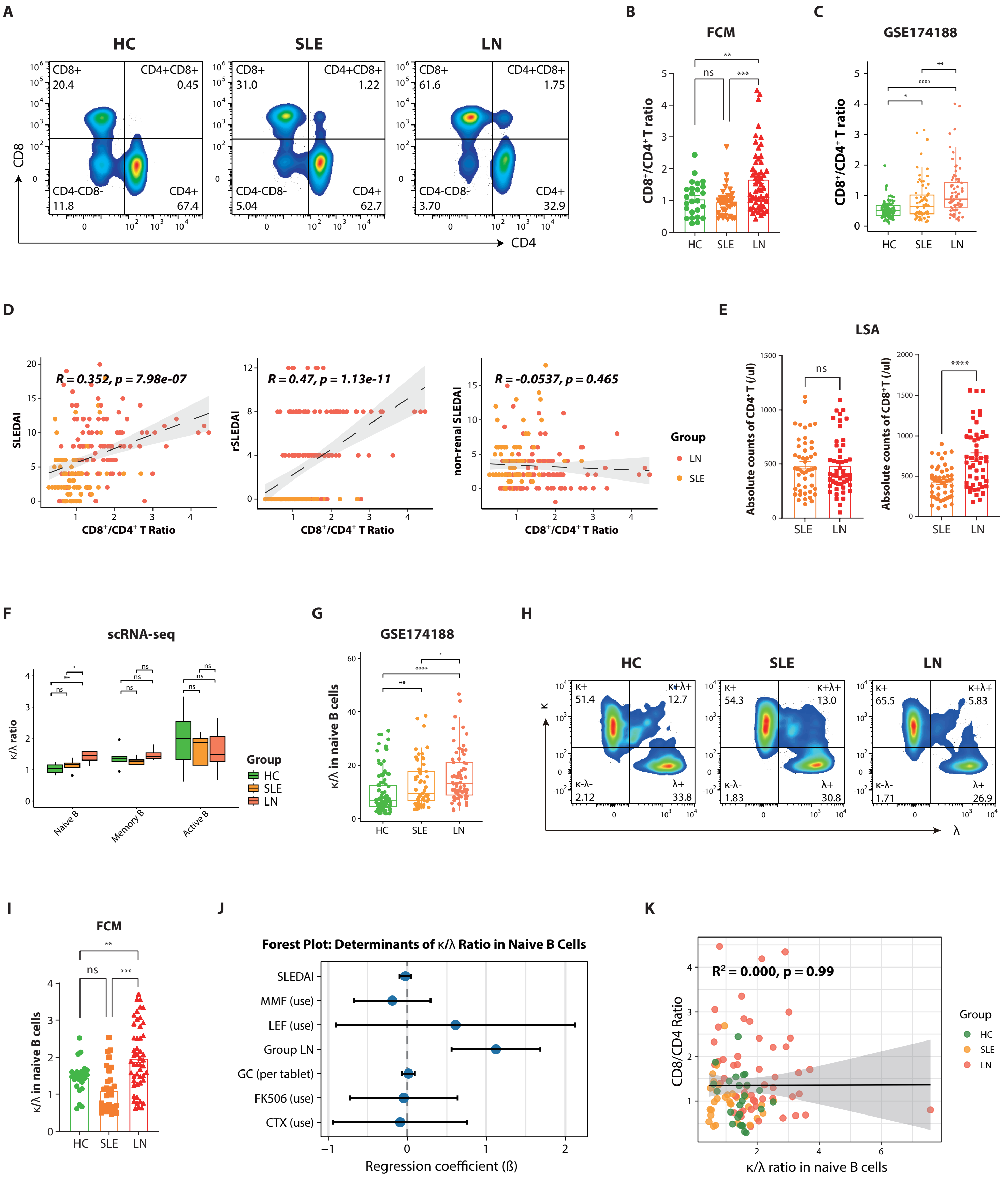

Supplementary Figure 4. The elevated $CD8^+/CD4^+$ T cell ratio and naive B cell κ/λ ratio in LN group.

A. The representative flow cytometry plots depict $CD8^+$ and $CD4^+$ T cells for each group.

B. The $CD8^+/CD4^+$ T cell ratio measured by FCM. Data are presented as mean ± SEM. 112 individuals were analyzed, including HCs (n = 25), SLE patients (n = 34) and LN patients (n = 53). Statistics were assessed by ANOVA and Fisher's least significant difference test. ns, not significant, $^{**}P < 0.01$, $^{***}P < 0.001$.

C. The $CD8^+/CD4^+$ T cell ratio (as defined by the original dataset) was determined using the independent validation cohort GSE174188, which included 99 HCs, 76 LN patients, and 68 SLE patients without LN. Box: IQR; line: median; whiskers: 1.5×IQR range from the quartiles; dots: individual samples. Significance levels: $^*p < 0.05$, $^{**}p < 0.01$, $^{****}p < 0.0001$.

D. Pearson correlation analysis was performed to assess the relationship between the $CD8^+/CD4^+$ T cell ratio and various SLEDAI scores in lupus patients. The $CD8^+/CD4^+$ T cell ratio was measured using both clinical laboratory-based LSA and FCM in a cohort of 187 patients, comprising 84 SLE patients without LN (SLE group) and 103 LN patients (LN group).

E. Comparison of absolute counts of $CD4^+$ T cells and $CD8^+$ T cells between SLE (n = 50) and LN (n = 50) were assessed through LSA from their clinical laboratory tests. Data are presented as mean ± SEM. The statistical significance of the differences was determined using an independent samples t-test. ns, not significant, $^{****}P < 0.0001$.

F. The κ/λ ratio in naive, memory, and active B cells across SLE, LN, and HC groups in the discovery cohort (n = 6 per group). Box: IQR; line: median; whiskers: 1.5×IQR range; outside points: outliers. Statistical significance was determined using the Mann-Whitney U test. Not significant (ns), $^*P < 0.05$, $^{**}P < 0.01$.

G. The naive B cell κ/λ ratio was computed in the independent validation cohort GSE174188 by first reassigning B cell subtypes using a custom reference dataset defined in this study. The recomputed ratio was then used for comparative analysis

among 99 healthy controls (HCs), 76 lupus nephritis (LN) patients, and 68 systemic lupus erythematosus (SLE) patients without LN. Box: IQR; line: median; whiskers: 1.5×IQR range from the quartiles; dots: individual samples. Significance levels: *p < 0.05, **p < 0.01, ****p < 0.0001.

H. Representative flow cytometry plots for each group, illustrating the distribution of κ^+ and λ^+ subpopulations within naive B cells ($CD3^-CD19^+CD27^-IgM^+$).

I. The ratio of κ-expressing to λ-expressing naive B cells measured by FCM. 70 individuals were analyzed, including HCs (n = 25), SLE patients (n = 30) and LN patients (n = 50). Data are presented as mean ± SEM. Statistics were assessed by ANOVA and Fisher's least significant difference test. ns, not significant, **P < 0.01, *** P < 0.001.

J. Forest plot from multivariable linear regression analysis evaluating clinical determinants of the κ/λ ratio in naive B cells (n = 80). The model was adjusted for daily glucocorticoid dose (GC, per tablet), use of mycophenolate mofetil (MMF), leflunomide (LEF), tacrolimus (FK506) and cyclophosphamide (CTX), and disease activity (SLEDAI). Symbols represent regression coefficients (β) with 95 % confidence intervals (horizontal lines); the vertical dashed line indicates no association ($\beta = 0$). A significantly higher κ/λ ratio was observed in patients with LN (Group LN) compared with those without LN. No significant associations were detected for any immunosuppressive medication or for SLEDAI score.

K. Linear regression analysis was performed to examine the relationship between the κ/λ ratio in naive B cells and the $CD8^+/CD4^+$ T cell ratio in peripheral blood (n = 105; HC, n = 25; SLE, n = 30; LN, n = 50). The regression line accounts for less than 0.1% of the variance ($R^2 < 0.001$, $p = 0.99$), indicating no statistically significant linear association. Data points are color-coded by group. Both ratios were quantified by FCM.

Supplementary Figure 5

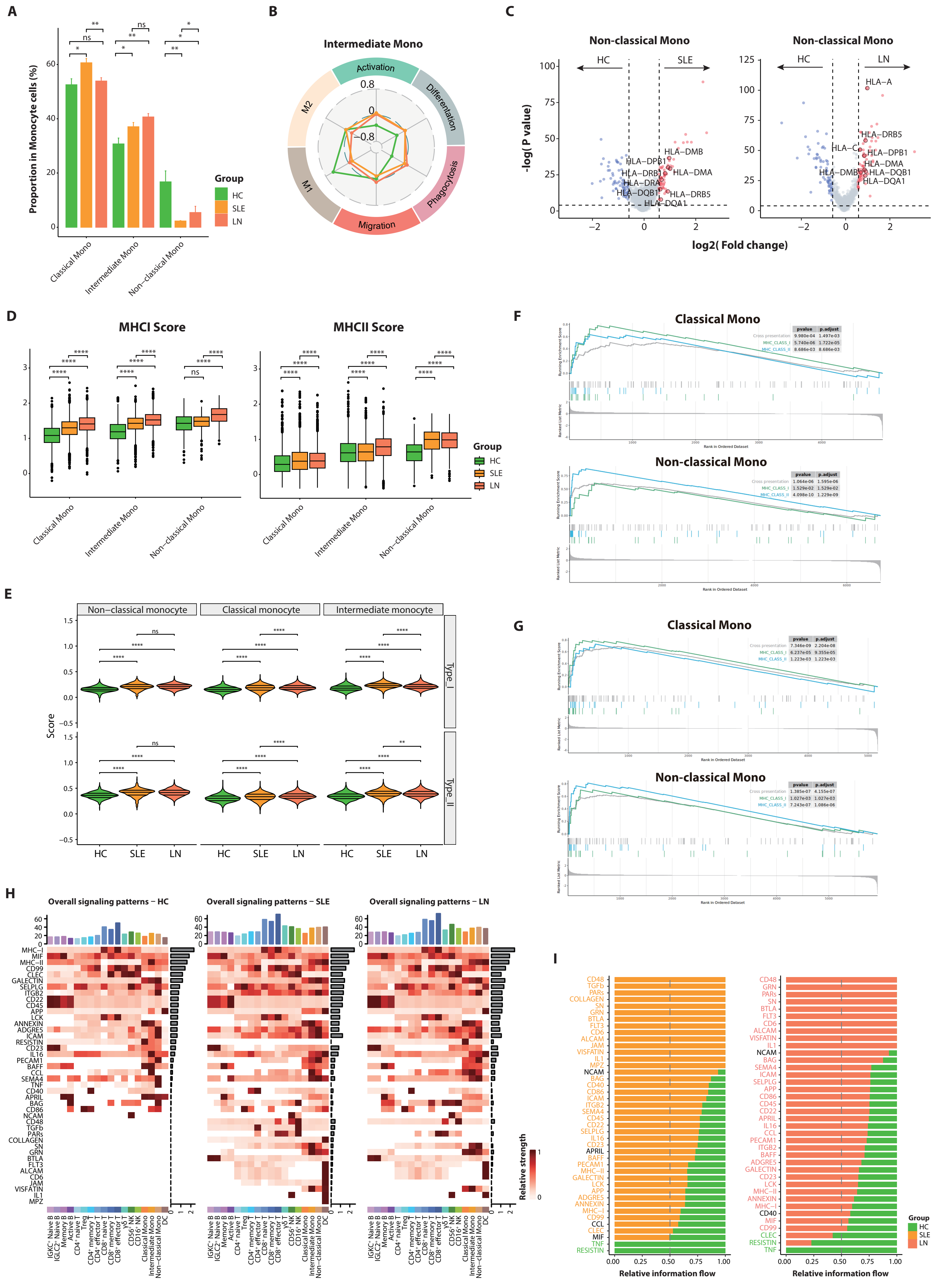

Supplementary Figure 5. Supplementary results for monocyte subclusters and cellular interactions.

A. Monocyte subcluster proportions across HC, SLE, and LN groups in the discovery cohort (n = 6 per group). Error bars represent the standard error of the mean. Significances were determined using the Mann-Whitney U test. ns, not significant, *P < 0.05, **P < 0.01.

B. Radar charts showing the average scaled module scores for functional gene sets in the intermediate monocyte subcluster, compared across HC, SLE, and LN groups from the discovery cohort.

C. Volcano plots showing differential gene expression in non-classical monocytes for SLE and LN groups compared to HC in the discovery cohort. The x-axis represents the log2 fold change of gene expression, while the y-axis shows the negative logarithm of the P-value.

D. Boxplots show module scores of MHC-I and MHC-II gene sets in monocyte subclusters from the discovery cohort. Box: IQR; line: median; whiskers: 1.5×IQR range; outside points: outliers. Statistical significances were determined using the Mann-Whitney U test. ns, not significant, ****P < 0.0001.

E. Violin plots show type I and II interferon-response gene module scores in monocyte subclusters from HC, SLE, and LN groups in the discovery cohort. Statistical significance was assessed using the Mann-Whitney U test for group comparisons. ns, not significant; **P < 0.01; ****P < 0.0001.

F. GSEA plots depict antigen presentation-related functional gene sets in classical and non-classical monocytes in the discovery cohort. Genes were ranked based on gene expression fold change between SLE and HC groups in each monocyte subset. P values are estimated by permutation test. ns, not significant, *P < 0.05, ***P < 0.001.

G. GSEA plots depict antigen presentation-related functional gene sets in classical and non-classical monocytes from the discovery cohort. Genes were ranked based on gene expression fold change between LN and HC groups in each monocyte subset. P values are estimated by permutation test. ns, not significant, *P < 0.05, ***P <

0.001.

H. Heatmaps show signal intensities of all cell-cell interactions in HC, SLE and LN groups from the discovery cohort.

I. The relative information flow for signaling pathways is compared between SLE and HC (left), and between LN and HC (right) in the discovery cohort. The significance of the observed differences in information flow is visually represented through color-coding and emphasized based on the respective group.

Supplementary Figure 6

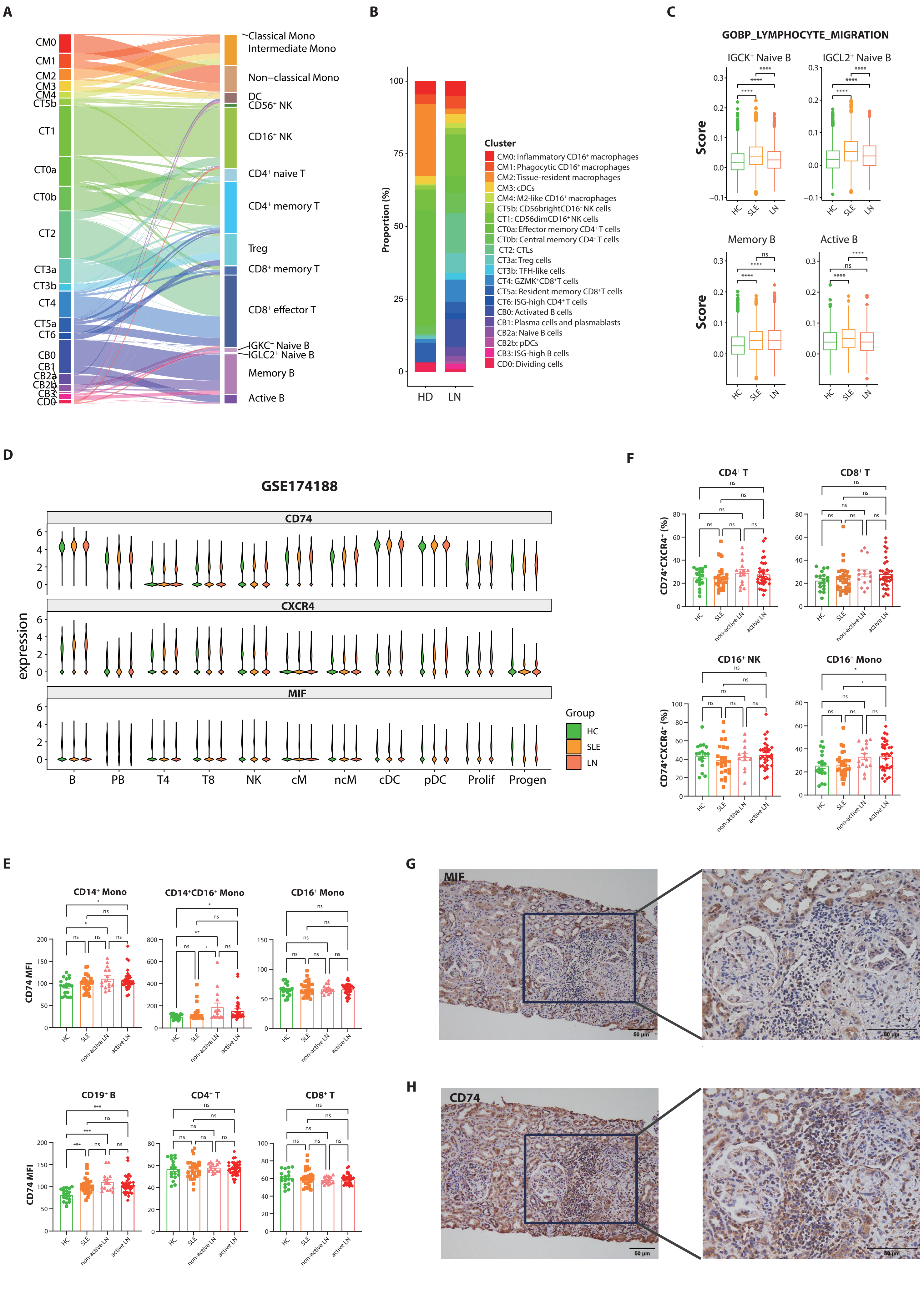

Supplementary Figure 6. The expression of MIF-(CD74+CXCR4) axis in circulating immune cells and renal cells.

A. Cell type prediction analysis of published scRNA-seq data from leukocytes isolated from kidney biopsy samples. This river plot provides a visual representation of the alignment between the original cell type annotations and the results of our predictive analysis.

B. Cell type composition of leukocytes isolated from kidney biopsy scRNA-seq data, according to the cell type definitions in the original dataset. Bar plots show the cell type composition estimated with original definition.

C. Module scores of lymphocyte migration related functional gene sets in each B cell subcluster from the discovery cohort. Box: IQR; line: median; whiskers: 1.5×IQR range; outside points: outliers. Statistical significances were determined using the Mann-Whitney U test. ns, not significant, ****$P < 0.0001$.

D. Expression of the MIF-(CD74+CXCR4) ligand–receptor pair in circulating immune-cell subsets from the independent validation cohort GSE174188.

E. Comparison of flow cytometry quantifying the mean fluorescence intensity (MFI) of CD74 across HC (n = 18), SLE (n = 29), non-active LN (n = 15) and active LN (n = 34) groups. Data are presented as mean ± SEM. Statistics were assessed by ANOVA and Fisher's least significant difference test. ns, not significant. *$P < 0.05$, **$P < 0.01$, ***$P < 0.001$.

F. The proportion of $CD74^{+}CXCR4^{+}$ in PB $CD4^{+}$ T cells, $CD8^{+}$ T cells, $CD16^{+}$ NK cells and $CD16^{+}$ monocytes across HC (n = 18), SLE (n = 29), non-active LN (n = 15) and active LN (n = 34) groups. Data are presented as mean ± SEM. Statistics were assessed by ANOVA and Fisher's least significant difference test. ns, not significant.

G. IHC staining for MIF in kidney biopsy from LN patient. Scale bar represents 50 μm.

H. IHC staining for CD74 in kidney biopsy from LN patient. Scale bar represents 50 μm.

Supplementary Table 1_Overview_of_scRNA-seq_cohort

Overview of the PBMC scRNA-seq discovery cohort			
	HC (n = 6)	SLE (n = 6)	LN (n = 6)
Age, years	34±9	33±8	35±10
Female	6(100)	6(100)	6(100)
SLEDAI	ND	7±1 *	11±4
rSLEDAI	ND	0 **	5±2
non-renal SLEDAI scores	ND	7±1	6±2
C3 (mg/dL)	ND	61.3 ± 15.6	56.4 ± 21.3
C4 (mg/dL)	ND	8.8 ± 4.3	13.4 ± 8.9
IgM (mg/dL)	ND	103 ± 46.7	85.9 ± 60.4
IgA (mg/dL)	ND	272.4 ± 114.4	249.3 ± 59.6
IgG (mg/dL)	ND	1571.7 ± 437.3	888.8 ± 663.3
eGFR (mL/min/1.73 m²)	ND	116 ± 15.8	107.5 ± 15.5
24hUTP (g)	ND	0.08 ± 0.03 *	3.6 ± 4.2
Hydroxychloroquine	0	6 (100)	5(83)
Mycophenolate mofetil	0	3(50)	2(33)
Cyclophosphamide	0	0	0
Tacrolimus	0	0	0
Azathioprine	0	0	0

Female sex and medications used in the last 6 months are shown as number of patients
SLEDAI, serum and urine measurements are shown as average ± standard deviation.
rSLEDAI : indication of proteinuria, pyuria, hematuria and/or casts in the SLEDAI score.
* $P < 0.05$, compared with LN
** $P < 0.01$, compared with LN

Source of the B cell-enriched PBMC scRNA-seq data				
ID	Age	Gender	Group	Data Source
P13	31	Female	lupus	GSE193867
P14	48	Female	lupus	GSE193867
P15	30	Female	lupus	GSE193867
HC7	34	Female	HC	GSE193867
HC8	43	Female	HC	GSE193867
HC9	42	Female	HC	GSE193867

Supplementary Table 2_Detailed clinical characteristics of the PBMC scRNA-seq discovery cohort

						Medications					Urine test				Renal Histopathology		
ID	Age	Gender	Group	SLEDAI	rSLEDAI	Glucocorticoid	MMF	Plaquenil	Scr（umol/L)	egfr-EPI	Urine sediment RBC(/HPF)	Urine sediment WBC(/HPF)	Casts	24hUTP (g)	ISN/RPS classification	Activity Index	Chronicity Index
P1	47	Female	SLE	8	0	8 tablets QD	-	0.2g BID	75	85.4	-	-	-	0.08815	-	-	-
P2	23	Female	SLE	8	0	2 tablets QD	0.75g BID	0.2g BID	48.8	131.9	-	-	-	0.0612	-	-	-
P3	28	Female	SLE	6	0	10 tablets QD	-	0.2g BID	60.8	121.3	-	-	-	0.081	-	-	-
P4	30	Female	SLE	8	0	10 tablets QD	0.75g BID	0.2g BID	57.8	121.3	-	-	-	0.055	-	-	-
P5	38	Female	SLE	8	0	4 tablets QD	-	0.2g BID	54.7	116.9	-	3-7	-	0.0897	-	-	-
P6	33	Female	SLE	6	0	4.5 tablets QD	0.5g BID	0.2g BID	58.1	118.9	-	-	-	0.1323	-	-	-
P7	23	Female	LN	9	4	2 tablets QD	0.5g BID	0.2g BID	73.4	104.52	2-6	3-5	-	2.145	Ⅳ+Ⅴ	8	7
P8	26	Female	LN	8	4	10 tablets QD	-	0.2g BID	41.3	134.8	-	-	-	11.3325	Ⅴ	0	0
P9	40	Female	LN	16	8	10 tablets QD	-	0.2g BID	68.6	100.66	17-23	4-9	-	0.4224	Ⅳ	6	2
P10	42	Female	LN	10	4	5 tablets QD	0.5g BID	0.2g BID	75.9	87.83	-	-	-	2.268	Ⅳ	13	7
P11	32	Female	LN	18	12	10 tablets QD	-	0.2g BID	68.5	106.67	15-20	4-6	-	5.3592	Ⅳ+Ⅴ	8	0
P12	49	Female	LN	8	4	6 tablets QD	-	-	56.5	110.27	7-13	-	-	0.2072	III	4	3
HC1	30	Female	HC	NA	NA	NA	NA	NA	NA	NA	NA	NA	NA	NA	NA	NA	NA
HC2	28	Female	HC	NA	NA	NA	NA	NA	NA	NA	NA	NA	NA	NA	NA	NA	NA
HC3	25	Female	HC	NA	NA	NA	NA	NA	NA	NA	NA	NA	NA	NA	NA	NA	NA
HC4	50	Female	HC	NA	NA	NA	NA	NA	NA	NA	NA	NA	NA	NA	NA	NA	NA
HC5	37	Female	HC	NA	NA	NA	NA	NA	NA	NA	NA	NA	NA	NA	NA	NA	NA
HC6	35	Female	HC	NA	NA	NA	NA	NA	NA	NA	NA	NA	NA	NA	NA	NA	NA

Supplementary Table 3_CellType_Count_By_Patient

ID	Group	Sorting	IGKC+ Naive B	IGLC2+ Naive B	Memory B	Active B	CD4 naive T	Treg	CD4 memory T	CD4 effector T	CD8 naive T	CD8 memory T	CD8 effector T	gd T	CD56 NK	CD16 NK	Classical Mono	Intermediate Mono	Non-classical Mono	DC	Platelet
HC1	HC	PBMC	53	35	131	22	1175	75	688	32	444	536	479	306	100	1005	982	669	97	12	65
HC2	HC	PBMC	118	97	228	14	961	125	777	236	599	450	1004	233	94	1050	575	351	66	11	89
HC3	HC	PBMC	584	422	528	38	1719	262	3729	200	1206	820	1458	300	177	3245	131	66	76	22	124
HC4	HC	PBMC	342	222	554	48	2197	200	3032	698	1021	861	1923	805	58	1912	225	144	114	38	153
HC5	HC	PBMC	240	183	235	33	1072	62	951	74	571	588	962	368	117	1949	79	41	16	2	23
HC6	HC	PBMC	145	104	172	10	1596	160	1473	108	667	945	827	420	83	542	188	103	97	12	52
HC7	HC	B cell-enriched PBMC	2197	1294	1159	180	0	0	0	0	1	0	0	0	0	0	0	0	0	0	2
HC8	HC	B cell-enriched PBMC	2539	1623	1942	190	13	2	5	1	0	0	0	0	0	1	3	0	14	15	0
HC9	HC	B cell-enriched PBMC	1618	1057	1073	59	6	1	3	0	0	0	0	0	0	0	0	0	1	0	0
P1	SLE	PBMC	60	51	149	20	281	253	643	48	394	167	691	39	72	230	999	649	43	47	23
P2	SLE	PBMC	1023	775	139	48	794	436	972	77	778	151	572	19	34	228	400	233	17	2	60
P3	SLE	PBMC	1250	856	660	164	650	356	1145	34	1036	225	254	51	15	69	233	168	7	3	31
P4	SLE	PBMC	313	270	185	9	207	135	623	31	223	129	231	17	15	87	3777	1690	114	94	35
P5	SLE	PBMC	866	378	389	45	736	299	492	128	1217	123	871	85	37	406	1288	779	54	6	222
P6	SLE	PBMC	471	391	234	25	1636	185	669	42	2116	187	621	76	49	110	1008	697	41	3	561
P7	LN	PBMC	408	287	196	24	1771	489	1340	30	2550	811	381	83	279	408	1173	839	408	62	76
P8	LN	PBMC	513	372	405	30	738	161	478	30	1596	129	351	31	29	71	1510	1123	51	28	28
P9	LN	PBMC	531	354	310	44	523	314	919	59	1533	296	759	113	30	107	954	739	59	2	37
P10	LN	PBMC	74	57	154	5	662	218	769	173	486	398	1828	126	28	304	1000	821	82	1	32
P11	LN	PBMC	540	339	514	58	422	207	524	120	864	291	1382	62	31	90	715	538	37	2	43
P12	LN	PBMC	241	150	199	48	843	164	584	101	324	131	770	128	43	286	727	522	42	5	133
P13	SLE	B cell-enriched PBMC	2464	1377	2175	486	2	2	5	0	5	0	0	0	0	0	0	0	0	15	0
P14	LN	B cell-enriched PBMC	1667	462	786	388	2	2	0	0	2	0	1	0	0	0	0	0	0	5	1
P15	LN	B cell-enriched PBMC	948	585	665	342	0	1	1	0	0	0	0	0	0	0	0	0	0	0	1

Supplementary Table 4_T_ExpandedClone_CellTypeCount

T_ExpandedClone_CellTypeCount_byGroup

Group	CD4 effector T	CD4 memory T	CD4 naive T	CD8 effector T	CD8 memory T	CD8 naive T	gd T	Treg
HC	407	166	1	2496	408	6	66	1
LN	235	21	0	2854	268	1	49	8
SLE	156	23	1	1832	84	0	65	0

T_ExpandedClone_CellTypeCount_byParticipant

ID	Group	CD4 effector T	CD4 memory T	CD4 naive T	CD8 effector T	CD8 memory T	CD8 naive T	gd T	Treg
HC1	HC	12	1	0	144	89	0	0	1
HC2	HC	149	5	0	515	21	0	31	0
HC3	HC	61	78	0	434	59	0	16	0
HC4	HC	134	78	1	636	104	6	7	0
HC5	HC	20	0	0	426	29	0	4	0
HC6	HC	31	4	0	341	106	0	8	0
P1	SLE	15	8	0	396	19	0	5	0
P2	SLE	60	2	0	356	16	0	6	0
P3	SLE	15	8	0	56	8	0	3	0
P4	SLE	18	5	1	85	10	0	3	0
P5	SLE	34	0	0	511	5	0	3	0
P6	SLE	14	0	0	428	26	0	45	0
P7	LN	3	1	0	45	61	0	2	0
P8	LN	16	0	0	140	0	0	1	0
P9	LN	12	8	0	240	25	0	1	2
P10	LN	79	5	0	1180	93	1	18	6
P11	LN	72	5	0	900	74	0	24	0
P12	LN	53	2	0	349	15	0	3	0

Supplementary Table 5_Top 10 CD8+ Effector T Cell Clones with Highest Predicted Affinity for GLCTLVAML

	Group	ID	TRBV	β CDR3	TRAV	α CDR3	Prob
1	LN	P12	TRBV27	ASRLRTGSSYEQYF	TRAV21	AVSRYSTLTF	0.85
2	LN	P12	TRBV27	ASRLRTGSSYEQYF	TRAV19	ALSEATSGTYKYIF	0.85
3	SLE	P5	TRBV7-9	ASRPDRASYGYTF	TRAV38-1	AFMTATGANSKLTF	0.84
4	LN	P10	TRBV7-9	ASSLDTGDAGNTIYF	TRAV14/DV4	AMRRPSGGYNKLIF	0.84
5	LN	P9	TRBV7-9	ATQLDYRSDTQYF	TRAV27	AGEGRDNYGQNFVF	0.81
6	SLE	P1	TRBV15	ATSTSLGRDEQFF	TRAV24	AFRVTGNQFYF	0.81
7	LN	P10	TRBV27	ASSTLDRSLGEQYF	TRAV8-2	VVSVWIYNQGGKLIF	0.81
8	LN	P11	TRBV27	ASSLGTGIFNEQYF	TRAV14/DV4	AMREDSFSGAGSYQLTF	0.80
9	LN	P11	TRBV27	ASSLYPGAKNNEQFF	TRAV19	ALMGSGAGSYQLTF	0.80
10	LN	P10	TRBV15	ATSRGTVSYEQYF	TRAV17	ATDFFGNEKLTF	0.79
