# Supplementary material for: Single-cell profiling unveils nephritis-related circulating immunological signatures in systemic lupus erythematosus patients
Source: Commun Biol. 2026 Jan 5;9:155. doi: 10.1038/s42003-025-09431-8 (PMC12868680; doi:10.1038/s42003-025-09431-8)
Supplement: Supplementary file 2 — Description of Additional Supplementary files [file 42003_2025_9431_MOESM2_ESM.pdf]

Description of Additional Supplementary files

File name: Supplementary Data 1

Description: Defining marker genes for all identified cell clusters.

File name: Supplementary Data 2

Description: Source data behind Figure 1C.

File name: Supplementary Data 3

Description: Source data behind Figure 2B, 2E, 2G.

File name: Supplementary Data 4

Description: Gene sets used for single-cell module scoring.

File name: Supplementary Data 5

Description: Source data behind Figure 3A.

File name: Supplementary Data 6

Description: Clinical characteristics of the FCM validation cohort and corresponding results (source data for Fig. 4A, C, E, 6D).

File name: Supplementary Data 7

Description: Clinical characteristics of the LSA validation cohort.

File name: Supplementary Data 8

Description: Description: Source data behind Figure 4D.

File name: Supplementary Data 9

Description: Source data behind Figure 6A.
