# Supplementary material for: Single-cell profiling unveils nephritis-related circulating immunological signatures in systemic lupus erythematosus patients
Source: Commun Biol. 2026 Jan 5;9:155. doi: 10.1038/s42003-025-09431-8 (PMC12868680; doi:10.1038/s42003-025-09431-8)
Supplement: Supplementary file 4 — Reporting Summary [file 42003_2025_9431_MOESM4_ESM.pdf]

nature portfolio

Corresponding author(s): Xiangmei Chen

Last updated by author(s): Nov 27, 2025

Reporting Summary

Nature Portfolio wishes to improve the reproducibility of the work that we publish. This form provides structure for consistency and transparency in reporting. For further information on Nature Portfolio policies, see our Editorial Policies and the Editorial Policy Checklist.

Statistics

For all statistical analyses, confirm that the following items are present in the figure legend, table legend, main text, or Methods section.

n/a	Confirmed	
☐	☒	The exact sample size (*n*) for each experimental group/condition, given as a discrete number and unit of measurement
☐	☒	A statement on whether measurements were taken from distinct samples or whether the same sample was measured repeatedly
☐	☒	The statistical test(s) used AND whether they are one- or two-sided<br>*Only common tests should be described solely by name; describe more complex techniques in the Methods section.*
☐	☒	A description of all covariates tested
☐	☒	A description of any assumptions or corrections, such as tests of normality and adjustment for multiple comparisons
☐	☒	A full description of the statistical parameters including central tendency (e.g. means) or other basic estimates (e.g. regression coefficient) AND variation (e.g. standard deviation) or associated estimates of uncertainty (e.g. confidence intervals)
☐	☒	For null hypothesis testing, the test statistic (e.g. *F*, *t*, *r*) with confidence intervals, effect sizes, degrees of freedom and *P* value noted<br>*Give P values as exact values whenever suitable.*
☒	☐	For Bayesian analysis, information on the choice of priors and Markov chain Monte Carlo settings
☒	☐	For hierarchical and complex designs, identification of the appropriate level for tests and full reporting of outcomes
☐	☒	Estimates of effect sizes (e.g. Cohen's *d*, Pearson's *r*), indicating how they were calculated

Our web collection on statistics for biologists contains articles on many of the points above.

Software and code

Policy information about availability of computer code

Data collection	The scRNA-seq, scTCR-seq, and scBCR-seq data generated and analyzed in this study are available under Project Accession No. PRJCA030971 upon publication. Publicly available scRNA-seq and scBCR-seq data were downloaded from the GEO database (accession GSE193867 and GSE174188) , Genome Sequence Archive for human (accession HRA001149 and HRA003738) and the Single Cell Portal (AMP Phase 1).
Data analysis	Data were analyzed using GraphPad Prism (Version 9.0), R (Version 4.0.5) or Python (Version 3.9.12). The codes for the data analysis in this study are available from the corresponding author upon reasonable request.

For manuscripts utilizing custom algorithms or software that are central to the research but not yet described in published literature, software must be made available to editors and reviewers. We strongly encourage code deposition in a community repository (e.g. GitHub). See the Nature Portfolio guidelines for submitting code & software for further information.

Data

Policy information about availability of data

All manuscripts must include a data availability statement. This statement should provide the following information, where applicable:

- Accession codes, unique identifiers, or web links for publicly available datasets
- A description of any restrictions on data availability
- For clinical datasets or third party data, please ensure that the statement adheres to our policy

The sequencing data have been deposited in the Genome Sequence Archive in National Genomics Data Center, China National Center for Bioinformation / Beijing Institute of Genomics, Chinese Academy of Sciences (GSA-Human: HRA008899) that are publicly accessible at https://ngdc.cncb.ac.cn/gsa-human.

Research involving human participants, their data, or biological material

Policy information about studies with human participants or human data. See also policy information about sex, gender (identity/presentation), and sexual orientation and race, ethnicity and racism.

Reporting on sex and gender	Since systemic lupus erythematosus predominantly affects women of childbearing age, with a male-to-female ratio of approximately 1:9, our sequencing cohort was predominantly female based on the biological characteristics of the disease. However, the validation cohort included both sexes, consistent with this rationale.
Reporting on race, ethnicity, or other socially relevant groupings	This study does not involve the reporting or analysis of race, ethnicity, or other socially relevant groupings.
Population characteristics	For the scRNA-seq cohort, we enrolled 12 SLE patients and 6 healthy controls. The validation cohort comprised an additional 187 lupus patients and 25 healthy controls. Demographic and clinical characteristics are detailed in Tables S1 (sequencing cohort) and S6 (validation cohort).
Recruitment	Participants were recruited at the Chinese People's Liberation Army General Hospital. All provided written informed consent. Well-defined exclusion criteria were implemented during screening, and no significant self-selection bias was detected in the recruitment process.
Ethics oversight	Ethical approval for this study was obtained from the Ethics Committee of Chinese People's Liberation Army General Hospital (Approval Nos. S2019-095-01 and S2022-640-01). All procedures complied with the ethical standards outlined in the Declaration of Helsinki.

Note that full information on the approval of the study protocol must also be provided in the manuscript.

Field-specific reporting

Please select the one below that is the best fit for your research. If you are not sure, read the appropriate sections before making your selection.

☒ Life sciences ☐ Behavioural & social sciences ☐ Ecological, evolutionary & environmental sciences

For a reference copy of the document with all sections, see nature.com/documents/nr-reporting-summary-flat.pdf

Life sciences study design

All studies must disclose on these points even when the disclosure is negative.

Sample size	Sample size determination drew on established protocols in the field. We enrolled 18 participants for single-cell analysis: 6 healthy controls, 6 SLE patients without nephritis, and 6 lupus nephritis patients. For validation, we leveraged two external scRNA-seq dataset (GSE193867 and GSE174188) from GEO and recruited an additional 187 lupus patients with 25 healthy controls. This approach balanced statistical requirements with practical constraints (e.g., sample availability). Group sample sizes provide adequate power for detecting biologically relevant insights in this exploratory study.
Data exclusions	No data were excluded; all participants' data were included in the final analyses.
Replication	Experimental reproducibility was ensured through sufficient biological replicates in all study phases.
Randomization	Participants were grouped according to disease status. As no interventions were administered, random allocation to study groups was neither required nor performed.
Blinding	The blinding procedure was not required in this study as no interventions were performed on the participants.

Reporting for specific materials, systems and methods

We require information from authors about some types of materials, experimental systems and methods used in many studies. Here, indicate whether each material, system or method listed is relevant to your study. If you are not sure if a list item applies to your research, read the appropriate section before selecting a response.

Materials & experimental systems

n/a	Involved in the study	
☐	☒	Antibodies
☒	☐	Eukaryotic cell lines
☒	☐	Palaeontology and archaeology
☒	☐	Animals and other organisms
☐	☒	Clinical data
☒	☐	Dual use research of concern
☒	☐	Plants

Methods

n/a	Involved in the study	
☒	☐	ChIP-seq
☐	☒	Flow cytometry
☒	☐	MRI-based neuroimaging

Antibodies

Antibodies used	The following antibodies were used in this study:<br>Flow cytometry: CD3-BV605 (BioLegend, cat# 317321), CD4-BV510 (BioLegend, cat# 300545), CD8-PE (BioLegend, cat# 344705), CD19-APC/Cy7 (BioLegend, cat# 302218), CD16-FITC (BioLegend, cat# 302006), CD14-PE/Cy7 (BioLegend, cat# 301814), CD74-APC (BioLegend, cat# 326811), CD27-BV510 (BioLegend, cat# 356420), IgM-APC (BioLegend, cat# 314510), CXCR4-BV421 (BioLegend, cat# 306518), and anti-Human κ/λ Light Chains (DAKO, cat# FR481).<br>Other applications: Anti-MIF (Abcam, cat# ab65869), anti-CD74 (Abcam, cat# ab270265), anti-CD19 (Abcam, cat# ab134114), anti-CD8 (Abcam, cat# ab237709), anti-CD4 (Abcam, cat# ab288724), anti-CD16 (Abcam, cat# ab246222), and anti-CD206 (Abcam, cat# ab64693).
Validation	All antibodies used in this study were validated by the manufacturers for their respective applications.

Clinical data

Policy information about clinical studies

All manuscripts should comply with the ICMJE guidelines for publication of clinical research and a completed CONSORT checklist must be included with all submissions.

Clinical trial registration	This study did not constitute a clinical trial and thus has no clinical registration number.
Study protocol	As this was not a clinical trial, no clinical trial protocol was followed.
Data collection	Data were retrospectively collected from patient medical records at the Chinese People's Liberation Army General Hospital.
Outcomes	No primary or secondary outcomes were predefined, as the study focused exclusively on single-cell analysis and validation.

Plants

Seed stocks	NA
Novel plant genotypes	NA
Authentication	NA

Flow Cytometry

Plots

Confirm that:

☒ The axis labels state the marker and fluorochrome used (e.g. CD4-FITC).

☒ The axis scales are clearly visible. Include numbers along axes only for bottom left plot of group (a 'group' is an analysis of identical markers).

☒ All plots are contour plots with outliers or pseudocolor plots.

☒ A numerical value for number of cells or percentage (with statistics) is provided.

Methodology

Sample preparation	Fresh blood samples were collected in EDTA-anticoagulated tubes. Peripheral blood mononuclear cells (PBMCs) were isolated by density gradient centrifugation using Ficoll-Paque™ PLUS (Cytiva, cat#17144003), followed by incubation in red blood cell lysis buffer. After confirming >85% viability, PBMCs were cryopreserved in SuperKine™ Serum/Protein-Free Cell Freezing Medium (Abbkine, cat#BMU108-CN), stored at −80°C for ≥24 hours, and subsequently transferred to liquid nitrogen. For experiments, frozen PBMC aliquots were rapidly thawed at 37°C, washed in RPMI-1640 medium (centrifugation at 300 × g for 5 min), and subjected to dead cell removal. Cells were then incubated with FcR Blocking Reagent (Miltenyi Biotec, Germany; #130-059-901) for 10 minutes at 4°C prior to antibody staining in chilled staining buffer.
Instrument	Stained cells were analyzed on a Guava easyCyte system (Luminex, USA).
Software	Data were analyzed using FlowJo v10.8.1 software (BD Life Sciences, USA).
Cell population abundance	The abundance of CD74+CXCR4+ subpopulations across immune cell types as follows: classical monocytes (30-90%), intermediate monocytes (30-98%), non-classical monocytes (10-50%), CD19+ B cells (20-80%), CD4+ T cells (10-45%), CD8+ T cells (5-40%), and CD16+ NK cells (5-50%).
Gating strategy	Initial gating employed FSC/SSC to exclude debris, doublets, and dead cells. Immune subsets were defined as: naïve B cells ($CD3^-CD19^+CD27^-IgM^+$), switched memory B cells ($CD3^-CD19^+CD27^+IgM^-$), unswitched memory B cells ($CD3^-CD19^+CD27^+IgM^+$), double-negative B cells ($CD3^-CD19^+CD27^-IgM^-$), $CD4^+$ T cells ($CD3^+CD19^-CD4^+CD8^-$), $CD8^+$ T cells ($CD3^+CD19^-CD4^-CD8^+$), $CD16^+$ NK cells ($CD3^-CD19^-CD4^-CD8^-CD16^+$), classical monocytes ($CD14^{++}CD16^-$), intermediate monocytes ($CD14^+CD16^+$), and non-classical monocytes ($CD14^-CD16^{++}$).

☒ Tick this box to confirm that a figure exemplifying the gating strategy is provided in the Supplementary Information.
